# LASP1-S100A11 axis promotes colorectal cancer aggressiveness by modulating TGFβ/Smad signaling

**DOI:** 10.1038/srep26112

**Published:** 2016-05-16

**Authors:** Ya Niu, Ziyun Shao, Hui Wang, Jiaqi Yang, Feifei Zhang, Yuhao Luo, Lijun Xu, Yanqing Ding, Liang Zhao

**Affiliations:** 1 Department of Pathology, Nanfang Hospital, Southern Medical University, Guangzhou, China; 2 Department of Pathology, School of Basic Medical Sciences, Southern Medical University, Guangzhou, China; 3 Department of Medical Oncology, Affiliated Tumor Hospital of Guangzhou Medical University, Guangzhou, China

## Abstract

LIM and SH3 protein 1(LASP1) can promote colorectal cancer (CRC) progression and metastasis, but the mechanism remains unclear. Here, we show that LASP1 interacts with S100 calcium binding protein A11(S100A11) and enhances its expression in CRC. LASP1-S100A11 axis is essential for TGFβ-mediated epithelial-mesenchymal transition (EMT) and cell aggressive phenotype. Clinically, S100A11 is overexpressed in CRC tissues and localized in both the cytoplasm and the nucleus of CRC cells. Overexpression of S100A11 in cytoplasmic and nuclear subcellular compartments is associated with tumor metastasis and poor prognosis of CRC patients. Introduction of cytoplasmic and nuclear S100A11 promotes aggressive phenotypes of CRC cells *in vitro* as well as growth and metastasis of CRC xenografts, whereas suppressing S100A11 abrogates these effects. Furthermore, we identify flotillin-1 (FLOT1) and histone H1 as downstream factors for cytoplasmic and nuclear pathway of S100A11, which are required for LASP1-S100A11 axis-mediated EMT and CRC progression. These findings indicate S100A11, combined with LASP1, plays a critical role in promoting CRC metastasis via its subcellular effectors, FLOT1 and histone H1.

Colorectal cancer (CRC) is one of the most common malignancies worldwide and the leading cause of cancer deaths^1^. Clinically, metastasis is still the main cause of mortalities^2^,^3^, yet there is lack of effective strategies for its management. An initiating mechanism in the early stages of distant metastasis is the epithelial-mesenchymal transition (EMT), a complex process that permits a polarized epithelial cell to gain mesenchymal-cell like properties^4^. Cancer cells undergoing EMT acquire aggressive phenotypes and detach from the primary tumor mass, enter the surrounding stroma and migrate to the distant sites^5^. An increasing body of evidence from clinical and experimental studies has supported a role for EMT in CRC dissemination^6^.

LIM and SH3 protein 1 (LASP1) was initially identified from metastatic axillary lymph nodes of breast cancer patients. LASP1, a specific focal adhesion protein, is involved in several biological and pathological processes^7^,^8^,^9^. In our previous studies, the stimulation of classical EMT inducer TGFβ significantly increased the expression of LASP1^10^. Thus, LASP1 overexpression was frequently found in CRC tissues, especially in metastatic CRC tissues. Introduction of LASP1 induced EMT process and created aggressive phenotypes of cancer cells, thereby promoting cancer growth and metastasis^11^. Presently, it is urgent to uncover the molecular mechanism of LASP1 during cancer progression, which may contribute significantly to the development of new diagnostic strategies and potential drugs targets.

We have preliminarily identified S100 calcium binding protein A11 (S100A11) as a LASP1-modulated protein in human CRC. To date no study has systematically investigated the role of LASP1-S100A11 axis in CRC progression, or the molecular mechanisms by which S100A11 exerts its function. Thus, the current study was undertaken in order to determine the contribution of LASP1-S100A11 axis to aggressive CRC.

## Materials and Methods

### Cell culture and miRNA transfection

CRC cell lines LS174T, RKO, HT29, HCT116, SW480, and SW620 were obtained from the Cell Bank of the Chinese Academy of Sciences (Shanghai, China) and maintained as previously described^11^. All cells were authenticated by short tandem repeat (STR) profiling before receipt and were propagated for less than 6 months after resuscitation. Additionally, a human CRC cell subline with unique liver metastatic potential, designated as SW480/M5, was established in our laboratory^12^ and used in the analysis. All the cells were cultured in RPMI 1640 (Hyclone; Logan, Utah, USA) supplemented with 10% fetal bovine serum (FBS) (Gibco-BRL, Invitrogen; Paisley, UK) at 37 °C with a humidity of 5% CO_2_. Human recombinant TGFβ1 (Peprotech, London, UK) diluted with serum-free medium containing bovine serum albumin at a concentration of 10 ng/ml was used to stimulate the cells for 24 and 48 hours.

### Tumor tissue samples

Fresh primary CRC specimens and paired noncancerous colorectal tissue were provided by the Tumor Tissue Bank of Nanfang Hospital. In each case, a diagnosis of primary CRC had been made, and the patient had undergone elective surgery for CRC in Nanfang Hospital between 2007 and 2010. The pathological diagnosis was made in the Department of Pathology of Nanfang Hospital of Southern Medical University. The study was approved by the Ethics Committee of Southern Medical University and all aspects of the study comply with the Declaration of Helsinki. Ethics Committee of the Southern Medical University specifically approved that not informed consent was required because data were going to be analyzed anonymously.

### Animals

All animal experiments were carried out with the approval of the Southern Medical University Animal Care and Use Committee in accordance with the guidelines for the ethical treatment of animals. Nude *nu/nu* mice were maintained in a barrier facility in racks filtered with high-efficiency particulate air filter. The animals were fed with an autoclaved laboratory rodent diet. The mice in this study were purchased from the Experimental Animal Centre of Southern Medical University, which is certified by the Guangdong Provincial Bureau of Science. All animal experiments involved ethical and humane treatment under a license from the Guangdong Provincial Bureau of Science.

### Statistical analysis

Data were analyzed using SPSS version 19.0 software (SPSS; Chicago, USA). The Student *t*-test and the one-way ANOVA test were carried out for qRT-PCR. CCK-8 analysis was applied to calculate the tumor growth curve. Significance of correlation between the expression of S100A11 and histopathological factors were determined using Pearson’s chi-squared (χ^2^) test. Kaplan-Meier plots were used to estimate the prognostic relevance of S100A11 in univariate analysis. Multivariate analysis was performed by applying Cox proportional hazards test. Statistical significance was established at *P* < 0.05.

## Results

### LASP1 positively regulates S100A11 expression by protein interaction

**Figure 1:**
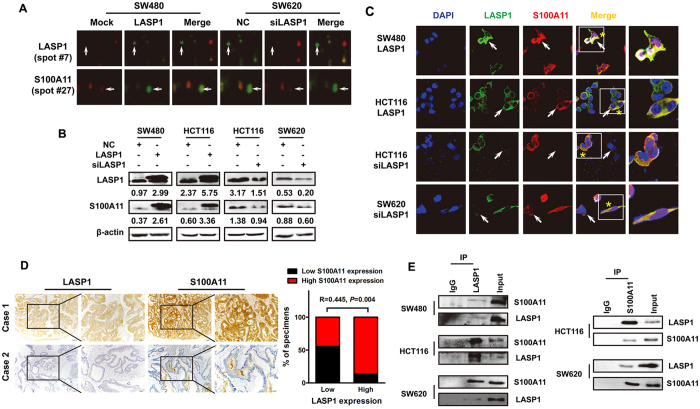
LASP1 regulates S100A11 expression by protein interaction. (**A**) 2-D DIGE was performed to screen the differentially expressed proteins in SW480/LASP-1 cells or SW620 cells that were transfected with LASP-1 siRNA and control cells. The enlarged images of two differentially expressed protein spots in DIGE analysis were shown. The protein spots are indicated (white arrows). (**B**) Western blot was performed to detect the expression of S100A11 and LASP1 protein in indicated cells. (**C**) The subcellular localization of S100A11 and LASP1 in indicated cells was assessed by immunofluorescence staining (original magnification ×2400). The arrow shows LASP1-overexpressing or LASP1-silenced CRC cells. The yellow signals of the box areas (yellow star) highlight the co-localization between LASP1 and S100A11. (**D**) Paraffin-embedded CRC sections were stained with anti-S100A11 or anti-LASP1 antibodies. Visualizations of two representative cases were shown. (**E**) Endogenous interaction between S100A11 and LASP1 in CRC cells. Cells were lysed and purified by anti-LASP1 or anti-S100A11 affinity gel; protein pellets were analyzed by western blot with anti-LASP1 or anti-S100A11. The full-length blots/gels are presented in Supplementary Fig. S4.

To explore the downstream molecules modulated by LASP1, we performed 2-D difference gel electrophoresis (2-D DIGE). Proteomic study revealed that one of the candidates LASP1-modulated proteins was identified as S100 calcium binding protein A11 (S100A11), which was positively correlated with LASP1 expression (Fig. 1A). The enhanced intensity of S100A11 was detected in LASP1-overexpressing SW480 cells, meanwhile the weakened intensity of S100A11 was observed in LASP1-silenced SW620 cells. Western blot and immunofluorescence assays obtained results that were consistent with the proteomic analysis (Fig. 1B,C). To further investigate the relationship between LASP1 and S100A11 in clinical tissue samples, immunohistochemistry assay were performed and showed that S100A11 expression was obviously correlated with LASP1 expression (Fig. 1D; R = 0.445, *P* = 0.004).

To address the regulatory mechanism of LASP1, we detected the effect of LASP1 on transcriptional activation and protein stability of S100A11. The results showed that LASP1 did not affect the expression of S100A11 mRNA and ubiquitin-mediated degradation of S100A11 (Supplementary Figs S1 and S2). Of note, we observed unambiguous co-localization of LASP1 and S100A11 protein in CRC cells (Fig. 1C). The protein interaction was also verified by further co-IP assay in protein extraction of CRC cells (Fig. 1E). This protein interaction, however, did not influence the expression of LASP1 protein (Supplementary Fig. S3).

### S100A11 is essential for LASP1-mediated EMT and cell aggressive phenotype

**Figure 2:**
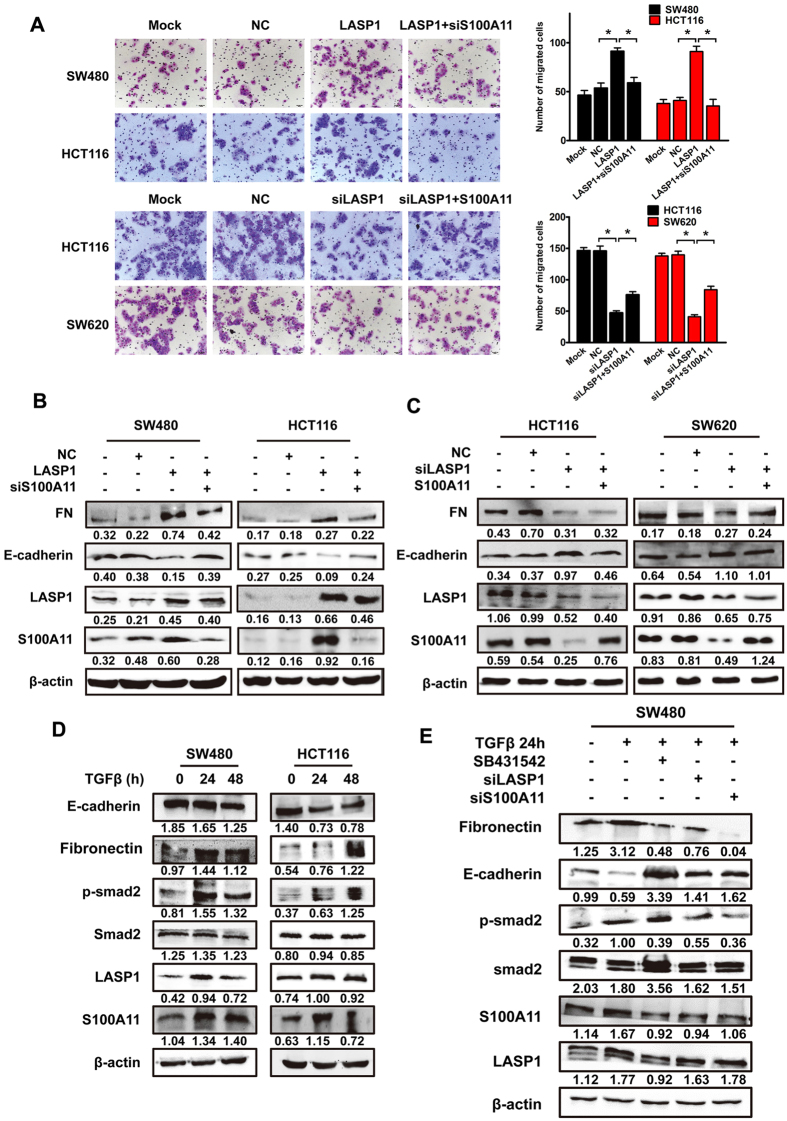
S100A11 is required for LASP1-mediated EMT and cell aggressive phenotype. (**A**) Representative figures and data of transwell assay for indicated cells. Each bar represented the mean ± SD. The results were reproduced in three independent experiments. The asterisk (*) indicates P < 0.05. (**B**) Western blotting analysis of EMT markers in indicated cells co-transfected with LASP1 vector and S100A11 siRNA. (**C**) Western blotting analysis of EMT markers in indicated cells co-transfected with LASP1 siRNA and S100A11 vector. (**D**) Western blotting analysis of EMT markers, phosphorylated Smad2, LASP1 and S100A11 in indicated cells in response to the treatment with 10 ng/mL TGFβ for 0, 24 and 48 hours. (**E**) Western blotting analysis of EMT markers and phosphorylated Smad2 in indicated cells co-treated with TGFβ and inhibitor SB431542, LASP1 siRNA or S100A11 siRNA. Representative figures were shown. The results were reproduced in 3 independent experiments. The full-length blots/gels are presented in Supplementary Fig. S6.

To address the pivotal role of LASP1-S100A11 axis in LASP1-mediated EMT and cell aggressive phenotype, we performed a rescued experiment to detect the expression of EMT markers and aggressive capacity of CRC cells. The results indicated that depletion of S100A11 weakens cell migration and reverses EMT induced by LASP1 in CRC cells, whereas restoring expression of S100A11 recovered aggressive capacity and renewed EMT process of CRC cells (Fig. 2A–C).

To elucidate the relationship between S100A11 and TGFβ signaling pathway, we analyzed the effect of recombinant TGFβ in CRC cells. The data demonstrated that TGFβ stimulation brings a significant change in classical EMT markers, such as E-cadherin and fibronectin (FN) (Fig. 2D). The cells displayed a spindle-shaped and fibroblastic-like phenotype instead of the cobblestone-like phenotype (Supplementary Fig. S5). As a result of TGFβ treatment, Smad2, a key mediator of TGFβ signaling, was activated through Smad2 phosphorylation (Fig. 2D). Meanwhile, we observed increased expression of LASP1 and S100A11 in response to TGFβ for 24 h and 48 h (Fig. 2D). Expectedly, TGFβ receptor inhibitor SB431542, LASP1 siRNA or S100A11 siRNA could suppress Smad2 phosphorylation and counteract EMT induced by TGFβ treatment, respectively (Fig. 2E).

### Both cytoplasmic and nuclear S100A11 is associated with poor prognostic phenotype of CRCs

**Figure 3:**
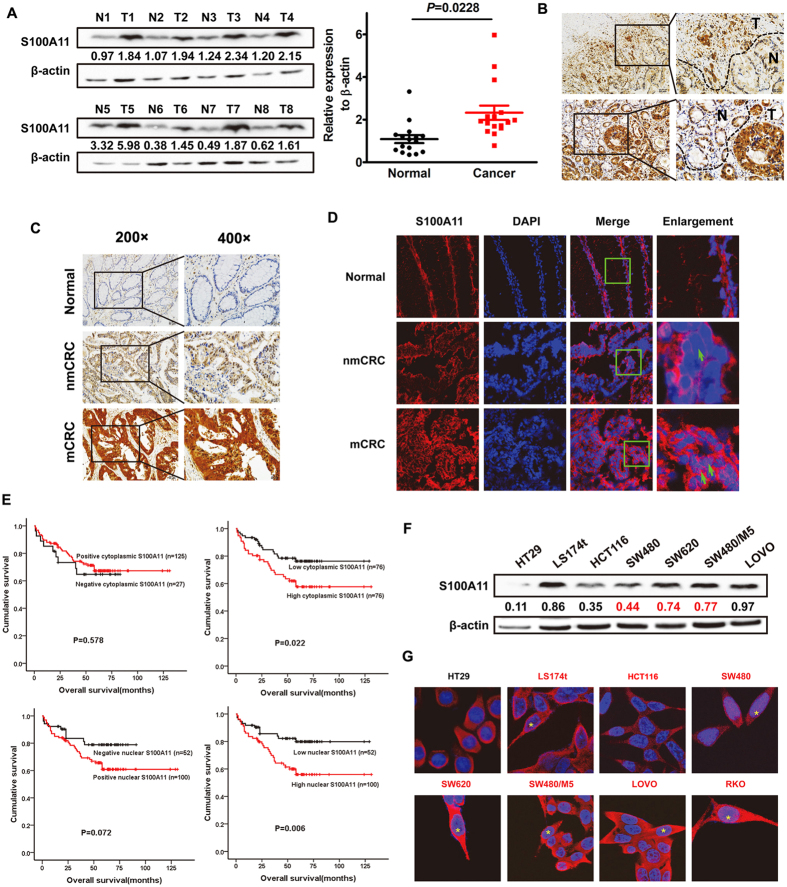
Both cytoplasmic and nuclear S100A11 is associated with poor prognostic phenotype of CRCs. (**A**) Western blot analysis of S100A11 in CRC tissues (T) and adjacent non-tumor tissues (N). The immunosignal was quantified using densitometric scanning software, and the relative protein abundance was determined by normalization with β-actin. (**B**) IHC analysis of S100A11 protein expression in CRC tissues (N) and adjacent non-tumor tissues (T). (**C**) IHC analysis of S100A11 protein expression in normal mucosa tissues, non-metastatic CRC (nmCRC) and metastatic CRC (mCRC) tissues. (**D**) IF analysis of S100A11 protein expression in normal mucosa tissues, nmCRC and mCRC tissues (original magnification ×2400). (**E**) Kaplan–Meier survival curves and univariate analyses (log-rank) for CRC patients with distinct expression level and subcellular localization of S100A11. (**F**) Western blot analysis for the expression of S100A11 in CRC cell lines. (**G**) IF analysis for subcellular localization of S100A11 in CRC cell lines (original magnification ×2400). The full-length blots/gels are presented in Supplementary Fig. S9.

To evaluate clinical significance of S100A11 protein, we detected the localization and expression of S100A11 and analyzed its relationship with pathological features. As shown in (Fig. 3A,B), S100A11 was up-regulated in CRC samples, compared to adjacent non-tumor tissues (*P* = 0.0228). Immunohistochemistry assay showed that S100A11 expression was detected in both cytoplasm and nucleus of benign and malignant epithelial cells. As shown in Supplementary Table S3, positive rate of cytoplasmic and nuclear S100A11 expression was significantly higher in CRC tissues, compared to adjacent non-tumor samples (*P* < 0.05). According to reclassification as described above, higher expression of S100A11 also frequently existed in CRC samples, especially in metastatic CRCs (*P* < 0.05; Fig. 3C,D). To further analyze its relationship with clinical features, both cytoplasmic and nuclear overexpression of S100A11 was closely related to lymph node metastasis and tumor recurrence of patients with CRC. Kaplan-Meier analysis displayed a correlation between heterogeneous localization of S100A11 overexpression and overall survival times of patients with CRC (Fig. 3E). Such a relationship observed in patients with high T stage (i.e., T3 and T4) was clearer than that in low T stage (i.e., T1 and T2) (Supplementary Fig. S7). Unfortunately, multivariate analysis failed to confirm S100A11 overexpression in subcellular compartments as an independent prognostic factor for CRC (Supplementary Table S4).

### Exogenous S100A11 localized to cytoplasm and nucleus contributes aggressive phenotypes and induces EMT *in vitro*

We detected the endogenous expression of S100A11 protein in all CRC cell lines. Relatively higher expression of S100A11 was found in SW620, SW480/M5 and Lovo cells with high metastatic potential, compared to HT29, HCT116 and SW480 cells. Interestingly, a gradually increasing trend of immunoreactivity was detected in three cell lines (SW480, SW620 and SW480/M5) derived from the same patient, along with an increase of metastatic potential (Fig. 3F). Except HT29 and HCT116 cells, nuclear localization of S100A11 was obviously observed in all CRC cells (indicated as yellow asterisks; Fig. 3G, Supplementary Fig. S8).

**Figure 4:**
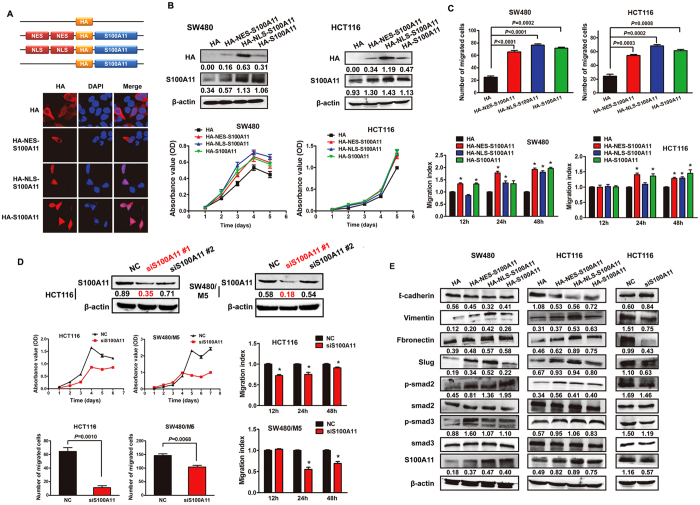
Cytoplasmic and nuclear overexpression of S100A11 contributes aggressive phenotypes and induces EMT *in vitro.* (**A**) HA-alone and three HA-tagged S100A11 proteins are depicted graphically and color coded. HA was fused to the N-terminus of the S100A11 cDNA. Exogenous NLS and NES tags were fused to the N-terminus of HA. Exogenous NLS and NES sequences target NLS-HA-S100A11 and NES-HA-S100A11 proteins to the nuclear and cytoplasmic subcellular compartments. IF assay was used to visualize subcellular localization of S100A11 in treated SW480 cells (original magnification ×2400). (**B**) SW480 and HCT116 cells were transiently transfected with HA, NES-HA-S100A11, NLS-HA-S100A11 and HA-S100A11 vectors. Western blot analysis was performed to detect the exogenous and total expression of S100A11 using anti-HA and anti-S100A11 antibodies, respectively (upper panel). The effect of S100A11 on cell proliferation was evaluated by CCK-8 assay (lower panel). (**C**) The invaded cells of transwell assay were counted under a microscope in five randomly selected fields. Bars represent the number of invaded cells (upper panel). Wound-healing assay was performed to evaluate the migratory ability. Bars represent migration index of treated or control cells. The distance migrated by treated cells was relative to that migrated by control cells (lower panel). (**D**) HCT116 and SW480/M5 cells were transiently transfected with S100A11 siRNAs. CCK-8 assay (lower left panel), transwell assay (upper right panel) and wound-healing assay (lower right panel) were used to evaluate proliferative and migratory captivity in the indicated cells. (**E**) Western blotting analysis of EMT markers in indicated cells treated with S100A11 vector or siRNA. Representative figures were shown. The results were reproduced in 3 independent experiments. The full-length blots/gels are presented in Supplementary Fig. S12.

To examine the effects of subcellular localization of S100A11 on CRC cell behaviors, either a duplicated nuclear localization sequence (NLS) motif or a duplicated nuclear export signal (NES) sequence was fused in-frame to amino-terminus of HA-S100A11 to generate NLS-HA-S100A11 or NES-HA-S100A11, respectively. Immunofluorescence assay revealed that the NLS-HA-S100A11 protein was exclusively present within the nucleus of SW480 cells. Conversely, the NES-HA-S100A11 protein was excluded from the nucleus. Finally, HA-S100A11 was expressed in both the cytoplasm and nucleus (Fig. 4A).

*In vitro* gain-of-function analyses were carried out using SW480 and HCT116 cells transiently transfected with recombinant expression vectors. Western blot analysis confirmed exogenous S100A11 overexpression in the transfected cells (Fig. 4B). CCK-8 assay revealed that exogenous S100A11, localized to cytoplasm and nucleus, enhanced the ability of cell proliferation (Fig. 4B). The migratory ability was promoted independently of nuclear or cytosolic expression, as assessed by transwell and wound-healing assays (Fig. 4C; Supplementary Fig. S10). In addition, the mesenchymal markers (vimentin and fibronectin) and transcription factor slug were upregulated, and the classical Smad pathway (smad2 and smad3) was activated, while epithelial marker E-cadherin was downregulated through introduction of S100A11 expression by transient transfection (Fig. 4E).

*In vitro* loss-of-function analyses were performed using siRNA-mediated RNA interference. We designed two siRNA against S100A11 and detected the efficiency of RNA interference by Western blot. The results showed that only #1 siRNA obviously decreased S100A11 expression. We selected the siRNA for further analysis. The opposite phenomenon was observed upon depletion of S100A11 on HCT116 and SW480/M5 CRC cells, with a relatively elevated S100A11 expression level (Fig. 4D; Supplementary Fig. S11).

### Subcellular localization of endogenous S100A11 overexpression promotes CRC growth and progression *in vivo*

**Figure 5:**
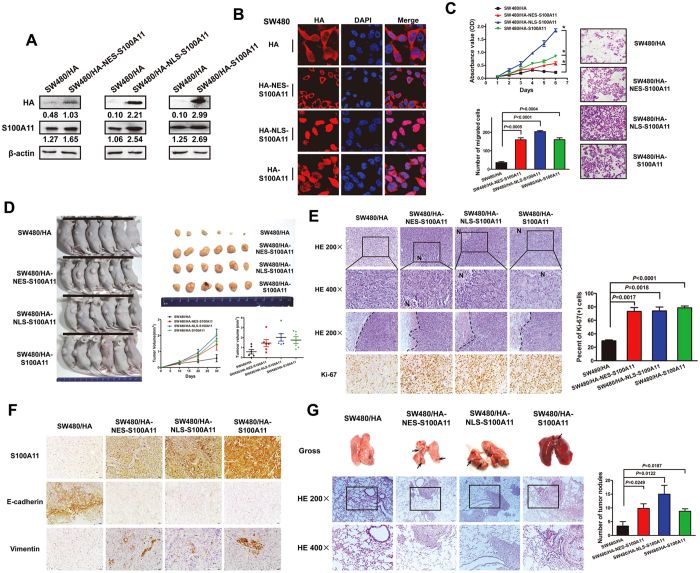
Subcellular localization of endogenous S100A11 overexpression promotes CRC growth and progression *in vivo.* (**A**) Western blot analysis was used to detect the endogenous overexpression of S100A11 in the stably subcellular transfectants. (**B**) IF assay was used to visualize subcellular localization of S100A11 in stable transfectants (original magnification ×2400). (**C**) CCK-8 and transwell assays were used to evaluate the ability of cell proliferation and migration in indicated cells. (**D**) Tumor cells were injected subcutaneously into the back of nude mice to evaluate tumorigenesis. Representative figure of tumors formed. Tumor volume in the back of nude mice injected with indicated cells was measured. The data of all primary tumors are expressed as mean ± SD. Scatter plots of tumor volume derived from indicated cells at 30 d after subcutaneous implantation. (**E**) The representative photographs of haematoxylin and eosin H&E staining of subcutaneous tumor are shown. Proliferative ability was measured by the Ki-67 proliferative labeling index. (**F**) Paraffin-embedded tumor sections were stained with with anti-S100A11, anti-E-cadherin or anti-vimentin antibodies. (**G**) Tumor cells were injected into nude mice through the tail vein to evaluate the lung homing potential of cells. The number of metastatic lung nodules in individual mice was counted under the microscope. The magnification areas indicated metastatic nodes in the lung. The full-length blots/gels are presented in Supplementary Fig. S15.

A causative role for subcellular localization of S100A11 in an experimental animal model was investigated by subcutaneous injection and tail vein injection of tumor cells. SW480 CRC cell lines with stable S100A11 overexpression targeting to cytoplasm and nucleus were established and successfully validated by Western blot and immunofluorescence assay (Fig. 5A,B; Supplementary Fig. S13). Expectedly, *in vitro* experiments confirmed that stably subcellular transfectants show an increase in cell proliferative and migratory ability, especially for NLS-transfectant (Fig. 5C). Subsequently, a subcutaneous tumor model was used to assess the ability of tumor genesis and growth. As shown in Fig. 5D,E, the tumors in SW480/NES-HA-S100A11, SW480/NLS-HA-S100A11 and SW480/HA-S100A11 groups grew faster and showed higher Ki-67 labeling index than those in the SW480/HA group. In the control group, tumors had a clear boundary with the surrounding regions. In contrast, the primary tumors that were derived from three stable transfectants showed infiltrative growth, thereby invading the surrounding fat and muscle tissues (Fig. 5E). IHC assay revealed that S100A11 overexpression targeting to the cytoplasm and nucleus enhanced expression of mesenchymal marker (vimentin) and decreased expression of epithelial marker (E-cadherin) in subcutaneous tumors (Fig. 5F; Supplementary Fig. S14).

To observe the effect of endogenous S100A11 localized to cytoplasm and nucleus on the potential of homing capacity, we injected cancer cells into nude mice through tail vein to observe lung nodules formation. Compared to control group, more and larger tumor nodules were found in the lung of mice with S100A11-overexpressing groups with cytoplasmic or nuclear localization (Fig. 5G).

### FLOT1 and histone H1 are respectively downstream factors for cytoplasmic and nuclear pathway of S100A11

**Figure 6:**
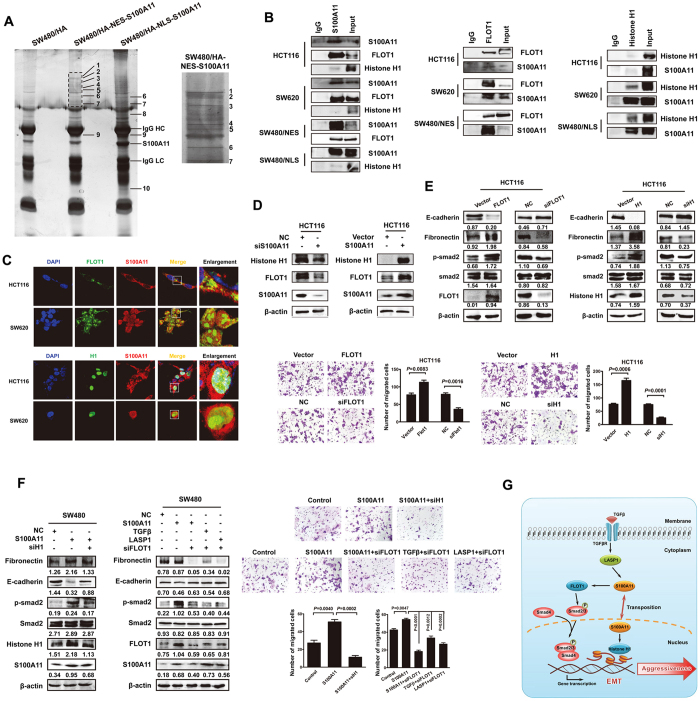
FLOT1 and histone H1 bind to S100A11 in cytoplasmic and nuclear compartment, respectively. (**A**) Immunoprecipitation of the whole proteins from SW480/HA-S100A11, SW480/NES-HA-S100A11 and SW480/NLS-HA-S100A11 cells with anti-HA antibody, respectively. Right panel showed partial enlarged view of lane 2. (**B**) Validation of endogenous interaction between S100A11 and FLOT1 or histone H1 in indicated cells. (**C**) The subcellular localization of FLOT1 or histone H1 and its co-localization with S100A11 in indicated cells was assessed by confocal microscopy (original magnification ×2400). (**D**) Western blot was performed to detect the expression of S100A11, FLOT1 and histone H1 protein in indicated cells. (**E**) Western blotting analysis of EMT markers in indicated cells treated with vector or siRNA of FLOT1 or histone H1 (upper panel). Representative figures were shown. The results were reproduced in 3 independent experiments. Representative figures and data of transwell assay for indicated cells were shown (lower panel). Each bar represented the mean ± SD. The results were reproduced in three independent experiments. (**F**) Western blotting analysis of EMT markers in indicated cells co-transfected with FLOT1 or histone H1 siRNA and S100A11 vector, TGFβ or LASP1 vector (left panel). Representative figures and data of transwell assay for indicated cells were shown (right panel). (**G**) A hypothetical model illustrating that LASP1-S100A11 axis contributes to the activation of the TGFβ/Smad pathway and multi-level promotes colorectal cancer aggressiveness. The full-length blots/gels are presented in Supplementary Fig. S18.

To identify the unknown downstream molecules of S100A11 function in cytoplasmic and nuclear compartment, the whole proteins of NES-HA-S100A11 and NLS-HA-S100A11 treated SW480 cells were extracted, immunoprecipitated with a specific HA-tag antibody, and resolved by SDS-PAGE. Differential protein bands were revealed by Coomassive blue staining in the resultant immunoprecipitate and analyzed by MS (Fig. 6A). The identified protein bands were summarized in Supplementary Table S5. Among them, band #4 was only present in SW480/NES-HA-S100A11 cells with cytoplasmic S100A11 overexpression and identified as Flotillin-1 (FLOT1). Band #10 was only detected in SW480/NLS-HA-S100A11 cells with nuclear S100A11 overexpression and identified as histone H1 (Supplementary Fig. S16). Both proteins were further confirmed by immunoprecipitation and immunoblot with anti-S100A11, anti-FLOT1 or anti-histone H1 antibody (Fig. 6B). Immunofluorescence assay verified subcellular localization of FLOT1 and histone H1, and their co-localization with S10011A in HCT116 and SW620 cells (Fig. 6C). The protein interactions upregulated expression of FLOT1 and histone H1 at posttranscriptional level (Fig. 6D), but did not influence the transcriptional activity of FLOT1 (Supplementary Fig. S17).

To further explore the function and role of FLOT1 and histone H1 in S100A11-mediated tumor progression, we performed gain- and loss-of-function assays. As expected, introduction of exogenous FLOT1 or histone H1 induced classical EMT process mediated by Smad pathway and increased the migratory capacity of CRC cells, whereas siRNA-mediated depletion of FLOT1 or histone H1 markedly reversed the process described above (Fig. 6E). To further explore the downstream mechanism of S100A11 in nucleus and cytoplasm of CRC cells, we observed the effect of abrogated FLOT1 or histone H1 expression on role of S100A11. The results showed that eliminated expression of nuclear histone H1 partly attenuated S100A11-induced EMT enhanced migration. Concordantly, reduced expression of cytoplasmic FLOT1 markedly neutralized the aggressive phenotypes mediated by TGFβ/LASP1/S100A11 axis in CRC cells (Fig. 6F).

## Discussion

LIM and SH3 protein 1 (LASP1) has been identified as a CRC metastasis-associated protein, which promotes CRC progression and leads to poorer clinical outcome^11^. TGFβ may activate Smad pathway and induce the classical EMT process by up-regulating LASP1 expression^10^. However, little is known about the exact mechanism underlying LASP1-mediated migration. In the current study, we identified S100A11 as a LASP1-modulated protein using a proteomic strategy based on 2-D DIGE combined with MS, and then validated the interaction of S100A11 with LASP1 in CRC cells. We analyzed the amino acid sequence of S100A11 and did not found proline-rich sequence in this protein. The previous studies have reported that NH_2_-terminal (amino acid residues 1–23) was identified as a phosphorylation site. Cytoplasmic S100A11 protein was specifically phosphorylated and bound to nucleolin on exposure of the cells to high Ca^2+^ or TGFbeta^13^,^14^. Althougth more works are needed to validate the hypothesis, we supposed that NH_2_-terminal of S100A11 might interact with SH3 domain in LASP1. The further study revealed that the formation of LASP1-S100A11 protein complex may contribute to stability of S100A11 and up-regulate S100A11 expression in CRC. When we abrogated LASP1 expression and removed the interaction, the expression of S100A11 will be recovered. Both LASP1 and S100A11 are essential for TGFβ-initiated EMT and migration, suggesting that the critical role of LASP1-S100A11 axis in CRC progression.

S100A11, previously named as S100C or Calgizzarin, belongs to the S100A11 family involved in the calcium signaling network, and regulates intracellular activities such as cell proliferation and motility, cell cycle progression, transcriptional regulation and cell differentiation^15^,^16^. Upregulated expression of S100A11 has been reported in several epithelial tumors and is linked to tumor metastasis^17^,^18^,^19^,^20^,^21^,^22^. Consistent with previous studies^19^,^23^,^24^, we demonstrated overexpression of S100A11 in CRC tissues and its relationship with lymph node metastasis of patients with CRC, suggesting that S100A11 might be an important onco-protein. To our knowledge, the current researches on S100A11 stay at the expression level. The functional role of S100A11 in CRC progression is still unknown.

It is also reported that S100A11 is associated with poor prognosis of cancer types, such as pancreatic and bladder cancer^18^,^21^, suggesting that S100A11 might be a novel predictive factor for poor prognosis. So far, however, no direct evidence has been shown for the predictive value of S100A11 in prognosis of CRC. In the current analysis, a significant correlation was found between S100A11 overexpression and worse clinical prognosis, and it was therefore possible that overexpression of S100A11 in the cancer cells resulted in more aggressive disease. Unfortunately, multivariate analysis showed that tumor differentiation, N classification and M classification, but not S100A11 expression, had positive predictive values for overall survival, implying the failure to identify overexpression of S100A11 as an independent prognostic factor. Therefore, further studies with a large sample size are needed to confirm these findings and to establish the role of S100A11A in the prognosis of overall survival of patients with CRC.

Our data provided strong evidences that S100A11 proteins existed not only in cytoplasm but also in nucleus of CRC cells. Not surprisingly, studies on S100A11 have until now focused on the role of S100A11 within the cytoplasm. However, the functional role of S100A11 in cytoplasm and nucleus of CRC cells remains unclear. Since we did not found the NLS sequence in S100A11 by analyzing the amino acid sequence of the protein, we suppose that S100A11 could move into nucleus through interaction with nuclear transporters. On exposure of the cells to high Ca^2+^ 13 or TGFβ^14^, cytoplasmic S100A11 protein was specifically phosphorylated, bound to nucleolin, and transferred to nuclei. Our findings have revealed that nuclear expression of S100A11 were frequently up-regulated in advanced CRC tissues or CRC cell lines with highly metastatic potential, suggesting the important role of S100A11 nuclear localization in CRC progression. We sought to directly address whether S100A11 localized to the nucleus (NLS-HA-S100A11), targeted to the cytoplasm (NES-HA-S100A11) or both subcellular compartments (HA-S100A11) displayed tumorigenic and aggressive properties. Our results indicated that both, nuclear and cytoplasmic-localized HA-S100A11, increased cell proliferation and migration, and promoted xenograft tumor growth and lung homing capacity in nude mice. Our data also showed relative higher proliferation effects with NLS-transfectant, which may result from higher transfection efficiency of NLS-HA-S100A11 vector. S100A11 targeted to the nucleus and/or cytoplasm activated Smad pathway and induced EMT, which is very similar to tumor-promoting properties of the upstream stimulatory factors LASP1 or TGFβ.

Despite the significant role of S100A11 in cancer progression, the downstream mechanism and molecular targets of S100A11 remains to be elucidated. Using our stable transfectants of S100A11 targeted to distinct subcellular compartments, we further explore the proteins interacting with S100A11 in the nuclear or cytoplasmic compartments of CRC cells via protein-protein interaction analysis. Among of the candidate proteins, histone H1 and flotillin-1 (FLOT1) were identified as new targets of S100A11 localized to the nucleus (NLS-HA-S100A11) and cytoplasm (NES-HA-S100A11), respectively. Knockdown of either histone H1 or FLOT1 partly counteracted the malignant phenotypes mediated by S100A11, suggesting their essential role in CRC progression mediated by TGFβ/LASP1/S100A11 axis.

Histone H1, one of the histone superfamilies, exists in somatic mammalian cells that bind to the linker DNA and stabilize the nucleosome particle contributing to higher order chromatin compaction. Nonetheless, it has been suggested that histone H1 plays a more dynamic and gene-specific role, participating in activation or repression of gene expression. Previous studies on the effect of H1 depletion on global gene expression have reported changes in the expression of small groups of genes, instead of affecting the vast majority of cellular genes^25^,^26^,^27^,^28^,^29^. However, the role of histone H1 in cancer remains to be defined. Indeed, depletion of histone H1 variants was reported to cause G1 arrest and inhibit cell proliferation in breast cancer^30^. Recently, it is demonstrated that histone H1 expression was related to Gleason pattern and proliferative index of prostate cancer. Silenced histone H1 significantly reduced cell proliferation in prostate cancer cells^31^. Our data provides new evidences that histone H1 induced EMT and promoted cell migration, suggesting its important role in CRC progression.

Flotillin-1 (FLOT1) is a plasma membrane lipid raft-localizing protein that is involved in internalization of membrane localizing proteins into the cytosol by endocytosis. Recently, FLOT1 was found to be associated with the development and progression in several types of cancer, including breast, renal, esophageal and lung carcinoma^32^,^33^,^34^,^35^. The lipid raft protein FLOT1 is up-regulated in esophageal squamous cell carcinoma (ESCC) cell lines and samples from patients and promotes ESCC cell proliferation and tumor growth in mice. FLOT1 activates tumor necrosis factor-α receptor signaling and sustains activation of NF-κB in ESCC cells^34^. FLOT1 also controls the malignant properties of neuroblastoma by regulating the endocytosis and degradation of membrane localizing ALK protein, suggesting that FLOT1 might contribute to the enhancement of oncogenic ALK signaling in neuroblastoma^36^. At present, there is only limited information about the involvement of FLOT1 in the oncogenicity and progression of CRC. Our results showed that FLOT1, identified as a downstream factor of S100A11, enhanced the malignant properties of CRC cells and involved in EMT mediated by TGFβ/LASP1/S100A11 axis. Interestingly, we first found nuclear localization of FLOT1 in SW620 cells (Fig. 6C). Further studies are needed to validate its localization and explore its biological significance. Taken together, our findings provide new insights into the contribution of FLOT1 to CRC progression, making FLOT1 an attractive therapeutic target.

Despite therapeutic advancements, current treatments against CRC remain challenging due to ineffective targeting of infiltrating CRC cells^37^. The TGFβ/Smad pathway has been considered as a therapeutic target for CRC^38^,^39^. In this context, however, given the opposing roles of the TGFβ/Smad pathway in tumor progression, to distinguish its tumor-suppressive role from the tumor-promoting potential in clinical therapy represents a challenge. Here, we demonstrated that LASP1-S100A11 axis was enhanced in CRC cells treated with TGFβ, which functionally promoted the CRC aggressiveness both *in vitro* and *in vivo*. Notably, cytoplasmic-localized S100A11 activated Smad pathway via upregulating FLOT1, whereas nuclear-localized S100A11 regulated gene expression via interacting histone H1. Above two distinct mechanisms jointly contributed to induce EMT process, which leads to CRC aggressiveness (Fig. 6G). Therefore, understanding the precise regulatory mechanism of LASP1-S100A11 axis in CRC progression will not only advance our knowledge of the pathogenesis of CRC, but also permit the development of novel diagnostic strategies and specific targeted drugs for managing the patients with advanced CRC.

## Additional Information

**How to cite this article**: Niu, Y. *et al*. LASP1-S100A11 axis promotes colorectal cancer aggressiveness by modulating TGFβ/Smad signaling. *Sci. Rep.* **6**, 26112; doi: 10.1038/srep26112 (2016).

## Supplementary Material

Supplementary Information
