# Supplementary material for: LASP1-S100A11 axis promotes colorectal cancer aggressiveness by modulating TGFβ/Smad signaling
Source: Sci Rep. 2016 May 16;6:26112. doi: 10.1038/srep26112 (PMC4867635; doi:10.1038/srep26112)
Supplement: Supplementary Information [file srep26112-s1.pdf]

Supplementary Information

LASP1-S100A11 axis promotes colorectal cancer aggressiveness by modulating TGFβ/Smad signaling

Ya Niu[1,2], Ziyun Shao[1,2], Hui Wang[3], Jiaqi Yang[2], Feifei Zhang[2], Yuhao Luo[2], Lijun Xu[2], Yanqing Ding[1,2], Liang Zhao[1,2]

1 Department of Pathology, Nanfang Hospital, Southern Medical University, Guangzhou, China

2 Department of Pathology, School of Basic Medical Sciences, Southern Medical University, Guangzhou, China

3 Department of Medical Oncology, Affiliated Tumor Hospital of Guangzhou Medical University, Guangzhou, China

Ya Niu, Ziyun Shao and Hui Wang contributed equally to this work.

Corresponding author at: Department of Pathology, Nanfang Hospital, Southern Medical University, Guangzhou, China. Tel./fax: +86 2061642148. E-mail address: liangsmu@foxmail.com (L. Zhao).

Supplementary Materials and Methods

RNA isolation, reverse transcription, and quantitative real-time PCR

Total RNA was extracted using Trizol reagent (Invitrogen). Total RNA was polyadenylated and underwent reverse transcription using PrimeScript™ RT Master Mix (TaKaRa, Dalian, China). Real-time PCR was carried out using a SYBR® Premix Ex Taq^{TM} II (TaKaRa, Dalian, China) on an ABI 7500HT system. GAPDH was used as an endogenous control. All samples were normalized to internal controls, and fold changes were calculated through relative quantification ($2^{-\Delta\Delta CT}$). Real-time PCR for target genes was performed as previously described.[1] The primers used are shown in Supplementary Table S1.

Western blot analysis

Protein expression was assessed by immunoblot analysis of cell lysates (20-60 μg) in RIPA buffer in the presence of rabbit antibodies to E-cadherin, mouse antibodies to β-catenin, fibronectin, vimentin, histone H1, β-actin (1:500; Santa Cruz, California, USA); and rabbit antibodies to N-cadherin, p-Smad2, Smad2, p-Smad3, Smad3, Slug (1:1000; CST, Danvers, MA); and rabbit antibody to S100A11, (1:1000; Proteintech, Chicago, IL); rabbit antibodies to flotillin-1 and HA (1:500; Abcam, Cambridge, UK). Bands were quantified by the densitometry function of the Quantity One software. β-actin was used as an endogenous control. All bands were normalized to internal controls, and fold changes were calculated through relative quantification. The data represented the average of the 3 independent experiments.

Immunofluorescence (IF)

Cells were cultured on coverslips overnight, fixed with 4% paraformaldehyde for 20 min and treated with 0.25% Triton X-100 for 10 min. After blocking in 10% normal blocking serum at room temperature for 10 min, slides were incubated with rabbit anti-S100A11 (1:400; Proteintech, Chicago, IL) and mouse anti-LASP1(1:100; Millipore, Billerica, MA) antibodies at 4°C overnight followed by washing with PBS three times. Coverslips were then incubated with fluorescein isothiocyanate (FITC)-conjugated and Texas Red (TR)-conjugated antibodies (1:120; Santa Cruz) for 30 min at room temperature, and then stained with 6-diamidino-2-phenylindole (DAPI; Invitrogen).

Proteomic analysis

Conventional 2-D electrophoresis analysis and MS identification were performed as previously described.[2] For 2-D difference gel electrophoresis (2-D DIGE), the proteins were labelled with fluorescent cyanine dyes (GE Healthcare, Milwaukee, Wisconsin, USA) following the manufacturer's instructions. In brief, 50 mg of extracted protein to be compared was labelled with 400 pmol Cy3 or Cy5, while 400 pmol Cy2 was employed to label 50 mg of internal standard protein of each sample at an equal amount. The labelling was performed on ice in the dark for 30 min, and then quenched with 1 ml of 10 mM lysine (Sigma) for 10 min. A 50 mg aliquot of Cy3- and Cy5-labelled samples was combined before mixing with 50 mg of Cy2-labelled internal standard. The 2× sample buffer with an equal volume (8 M urea, 2 M thiourea,

4% CHAPS, 2% Bio-lyte, pH 4-7, 130 mM dithiothreitol (DTT)) was added to the sample, and the final volume was adjusted to 450 ml with rehydration buffer (8 M urea, 4% CHAPS, 1% Biolyte, pH 4e7, 40 mM DTT). The proteins were applied to IPG (immobilised pH gradient) strips (pH 4-7, 24 cm) and focused on an IPGphor (GE Healthcare). Focused IPG strips were equilibrated, and then loaded onto 12% SDS-polyacrylamide gels (SDS-PAGE) using low-fluorescence glass plates on an Ettan DALT II system (GE Healthcare). All electrophoresis procedures were performed in the dark. The biological triplicates were run on three gels as analytical gels. In addition, another strip was performed in parallel as a preparative gel for picking spots as described in 2-D DIGE, except that the IPG strip was loaded with 1000 mg of proteins, and the gel was stained with Coomassie brilliant blue. After SDS-PAGE, the three analytical gels were scanned with a Typhoon 9410 scanner (GE Healthcare) with appropriate excitation/emission wavelengths specific for Cy2 (488/520 nm), Cy3 (532/580 nm) and Cy5 (633/670 nm) to generate nine protein spot maps.

DeCyder 5.0 software (GE Healthcare) was used for 2-D DIGE analysis according to the manufacturer's recommendation. The DeCyder differential in-gel analysis (DIA) module was used for pairwise comparisons of each sample with the internal standard in each gel. The DeCyder biological variation analysis (BVA) module was then used to simultaneously match all nine protein spot maps, using the Cy3/Cy2 and Cy5/Cy2 DIA ratios, to calculate average abundance changes and paired Student t test p values for the variance of these ratios for each protein pair across all

samples. The differential protein spots (|ratio|>2, $P<0.05$) which were altered consistently in all three protein spot maps were selected for further identification.

Immunohistochemistry (IHC)

Immunohistochemistry was performed, as previously described [3], to investigate the localization and expression of S100A11 in 152 human CRC tissues. The sections were incubated with primary antibodies against S100A11 (1:50) overnight at 4°C. Mayer's haematoxylin was used for nuclear counterstaining. The sections were mounted with a synthetic medium. The slides were reviewed by two or three pathologists blind to the study. To evaluate S100A11 expression levels, immunostained slides were evaluated using a method described previously [3,4]. Scores representing the percentage of positive cells were as follows: 0% (absent), 1-5% (sporadic), 6-25% (local), 26-50% (occasional), 51-75% (majority) and 76-100% (large majority). Intensity of staining of cancer cells was scored as 0 (no staining), 1 (weak staining, light yellow), 2 (moderate staining, yellowish brown), and 3 (strong staining, brown). An intensity score of ≥ 2 with at least 50% of LASP-1 positive cells was considered as having high expression (or over-expression), and <50% of LASP-1 positive cells or < 2 in intensity score was regarded as low expression. The discrepancies (< 5%) were resolved by simultaneous re-evaluation.

Nuclear S100A11-positivity was scored by determining percentage of positive nuclei regardless of cytosolic S100A11 expression and staining intensity. In analogy to the scoring of the proliferation marker Ki67 samples were considered as high nuclear expression if 10% or more cells showed nuclear staining for S100A11 [5].

Cell proliferation assays

Cell proliferation assays were carried out using Cell Counting Kit 8 (CCK8) (Dojindo; Kumamoto, Japan). Cells were plated in 96-well plates at a density of 1 × 10^4 cells per well and cultured in the growth medium. At the indicated time points, the number of cell in triplicate wells was measured using the absorbance at 450 nm of reduced WST-8 (2-(2-methoxy-4-nitrophenyl)-3-(4-nitrophenyl)-5-(2,4-disulfophenyl)-2H-tetrazolium ,monosodium salt).

Cell migration analysis

Cells from the serum-free medium (1 × 10^5 cells/100 μL) were added to the top chamber of each 8-mm–pore transwell chamber (Corning Star; Cambridge, Mass, USA). The bottom chamber was prepared using 10% FBS as a chemoattractant. Cells were allowed to migrate through the porous membrane for 24-48 h at 37°C. The cells that stuck to the lower surface of the membrane were treated with a fixation/staining solution (0.1% crystal violet, 1% formalin, and 20% ethanol) for visualization. The cells were counted under a microscope in 5 randomly selected fields (original magnification, ×200). At least 4 chambers from 3 different experiments were analyzed.

Wound healing assay

Confluent monolayers of cells were maintained in serum-containing growth medium for at least 6 d and then in serum-free medium for 24 h. A 200-μL plastic

pipette tip was used to scratch the monolayers. The wounded cells were then cultured in a serum-free medium for an additional 48 h and photographed under an inverted phase contrast microscope. Three different points were marked on the plate, and the distance between each point and the edge of the scratch wound was measured before and after cell migration. The mean migration distance (μm) was calculated by subtracting the length after 48 h from that at 0 h. The result was expressed as a migration index, ie, the distance migrated by treated cells compared with the distance migrated by control cells. Experiments were carried out in triplicate and repeated at least 5 times.

Plasmid constructs

All eukaryotic expression vectors were constructed in pEX-3 (pGCMV/MCS/Neo). Generation of nuclear export signals (NES) or nuclear localization signal (NLS)-tagged HA-S100A11 were achieved by using oligonucleotides encompassing either two NES or NLS sequences, an HA tag, and using these oligonucleotides in-frame to amino-terminus of pEX-3-S100A11 template by standard PCR techniques (Shanghai GenePharma Co., Shanghai, China). The sequences used to generate the recombinant vectors are as follows: HA tag (TAC CCA TAC GAC GTC CCA GAC TAC GCT), NLS sequence (CCA AAG AAG AAG AGA AAA GTG) and NES sequence (TTA GCA CTT AAA TTA GCT GGT TTG GAC ATA). The coding sequence for all NLS-HA-S100A11, NES-HA-S100A11 and HA-S100A11 constructs was verified by sequencing analysis.

Stable S100A11 over-expressing lines

For *in vivo* studies, the vector was transfected into CRC cell line, which had lower endogenous LASP-1 expression. Then, the standard cell culture medium was replaced with that containing 1 mg/ml G418. Stable cell lines, which respectively named as SW480/HA-S100A11, SW480/NES-HA-S100A11 and SW480/NLS-HA-S100A11, were selected after formation of resistant clones. For control cells, named as SW480/HA cells, recombinant vectors was replaced by control vector.

siRNA-mediated gene silencing

Expression of human S100A11, LASP1, FLOT1 and histone H1(0) was knocked down with siRNA duplexes targeting the sequence. The siRNAs were designed and chemically synthesized (Shanghai GenePharma Co., Shanghai, China) for targeting different coding regions of the genes. The negative control siRNA targeting unknown mRNA sequence was used as a control. The sequences used are shown in Table S2 (see supplementary material). Both siRNAs were synthesized from GenePharma (Shanghai, China). A BLAST search of the human genome verified that the selected sequences were specific for the target genes. Exponential growth phase cells were plated in 6-well plates at a density of 0.5×10^5 cells/ml, cultured for 24 h and transfected with 1 μg siRNA in reduced serum medium (OPTI-MEM-I) according to the manufacturer's protocol in 30-50% confluence. Fluorescein (FAM)-labeled negative control siRNA was used to visualize the transfection efficiency.

Tumor growth assay

For the tumor growth assay, 1×10^6 stable transfected cells were independently

injected subcutaneously into the left back of nude mice (n = 6/group). The tumor volume was calculated using the following formula: $V = 0.5 \times D \times d^2$, where V represents volume, D represents the longitudinal diameter, and d represents the latitudinal diameter.

Tumor metastasis assays

To determine the lung homing potential of cancer cells in vivo, we injected 5×10^6 stable transfected cells into nude mice (n = 8/group) through the tail vein. The mice were all sacrificed 8 weeks later, at which time individual organs were removed and metastatic tissue was analyzed using hematoxylin and eosin (H&E) and immunohistochemical (IHC) staining.

Co-immunoprecipitation (Co-IP)

The cells were transiently or stably transfected with the indicated constructs. Cells were harvested and lysed in 1 ml of lysis buffer (50 mM HEPES, 150 mM NaCl, 1 mM EDTA, 0.5% Nonidet P-40). Resulting lysates were subjected to immunoprecipitation with antibodies directed to the epitope tag. Immunoprecipitates were washed in lysis buffer, resolved by SDS-polyacrylamide gel electrophoresis, and subsequently analyzed by mass spectrum or protein immunoblotting.

Digestion of Proteins and Identification by Matrix-assisted Laser Desorption/ionization Time of Flight Mass Spectrometry

The identified protein bands in the gel were excised from the gel and were in-gel digested. Briefly, the gel bands were destained in 30 mmol/L $K_3Fe(CN)_6$ and 100

mmol/L $Na_2S_2O_3$, then dehydrated with 100% acetonitrile and dried in a stream of nitrogen gas. The dried gel pieces were incubated in a digestion solution consisting of 25 mM NH_4HCO_3 and 12.5 μg/ml trypsin (Promega, USA) for 16–18 h at 37°C. The tryptic peptide mixture was extracted and mixed with matrix a-cyano-4-hydroxycinnamic acid (CHCA) for mass spectrum analysis. Mass spectra results were obtained using an Applied Biosystems Voyager System 4800 matrix-assisted laser desorption/ionization time of flight mass spectrometry (MALDI-TOF) mass spectrometer (ABI, USA) with an accelerating voltage of MS/MS 8000 V. Mass fingerprinting was used for protein identification from tryptic fragment sizes in the NCBInr database (http://www.matrixscience.com) and SWISS-PROT database (http://web.expasy.org/docs/swiss-prot_guideline.html) with the MASCOT search engine for information such as protein name, mass score, and peptide match.

References

1 Wang, H. *et al.* miR-133a represses tumour growth and metastasis in colorectal cancer by targeting LIM and SH3 protein 1 and inhibiting the MAPK pathway. *Eur J Cancer* **49**, 3924-3935, doi:10.1016/j.ejca.2013.07.149 (2013).

2 Zhao, L., Wang, H., Li, J., Liu, Y. & Ding, Y. Overexpression of Rho GDP-dissociation inhibitor alpha is associated with tumor progression and poor prognosis of colorectal cancer. *Journal of proteome research* **7**, 3994-4003, doi:10.1021/pr800271b (2008).

3 Zhao, L. *et al.* Promotion of colorectal cancer growth and metastasis by the LIM and SH3 domain protein 1. *Gut* **59**, 1226-1235, doi:10.1136/gut.2009.202739 (2010).

4 Coppola, D. *et al.* Correlation of osteopontin protein expression and pathological stage across a wide variety of tumor histologies. *Clin Cancer Res* **10**, 184-190 (2004).

5 Tan, P. H. *et al.* Immunohistochemical detection of Ki67 in breast cancer correlates with transcriptional regulation of genes related to apoptosis and cell death. *Mod Pathol* **18**, 374-381 (2005).

Supplementary Figures

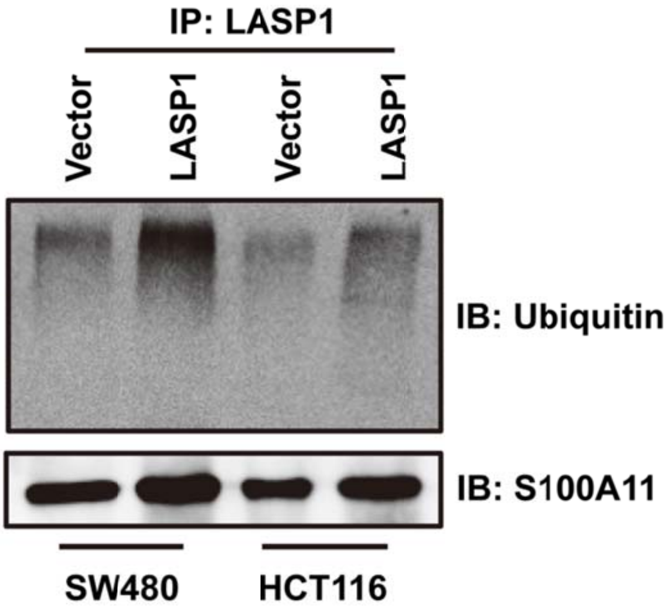

Figure S1, related to Figure 1. LASP1 did not induce ubiquitin-mediated degradation of S100A11. SW480 and HCT116 cells were transfected with LASP1 vector and harvested for ubiquitination assay. Introduction of LASP1 did not affect S100A4 ubiquitination-mediated degradation.

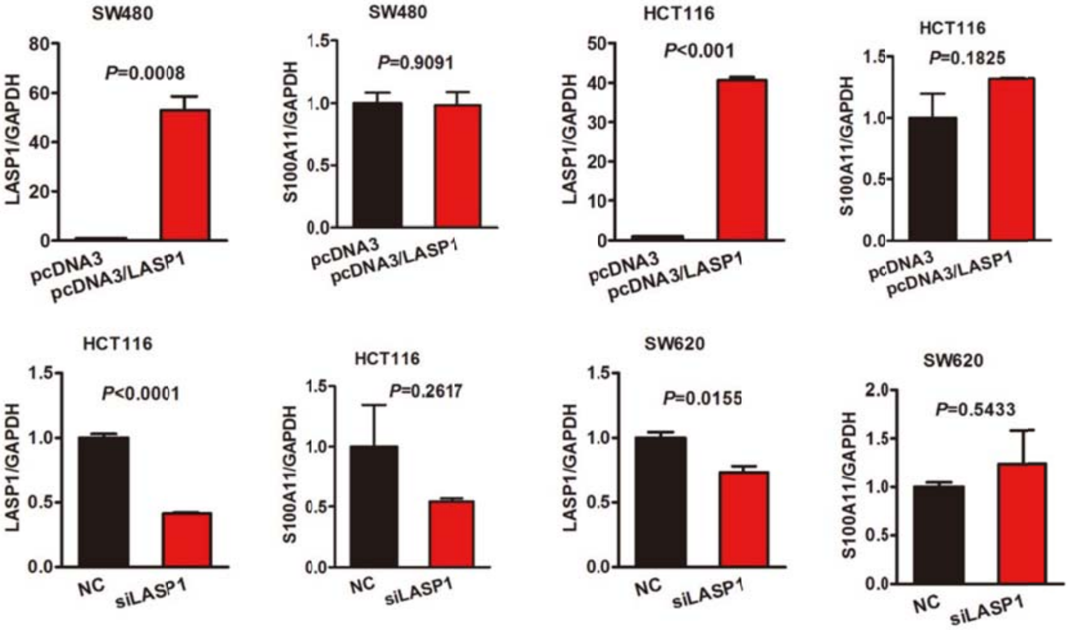

Figure S2, related to Figure 1. The effect of LASP1 expression on S100A11 mRNA expression. RT-PCR assay was performed to detect the expression of S100A11 and LASP1 mRNA in indicated cells.

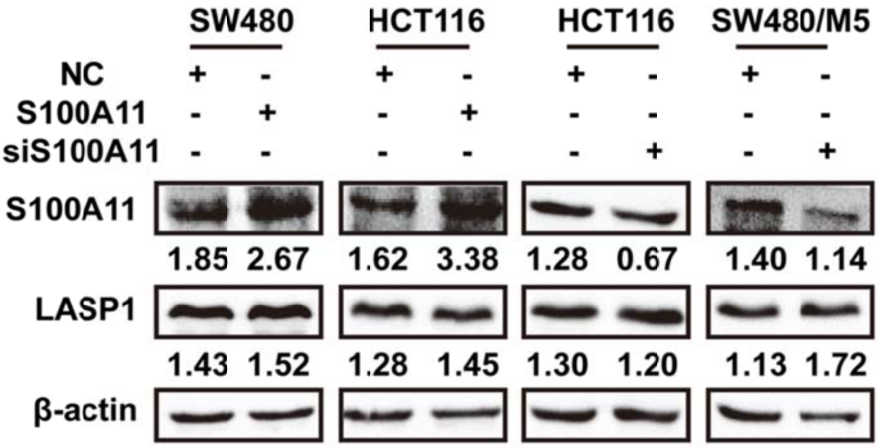

Figure S3, related to Figure 1. The effect of S100A11 expression on LASP1 protein expression. Western blot was performed to detect the expression of S100A11 and LASP1 protein in indicated cells.

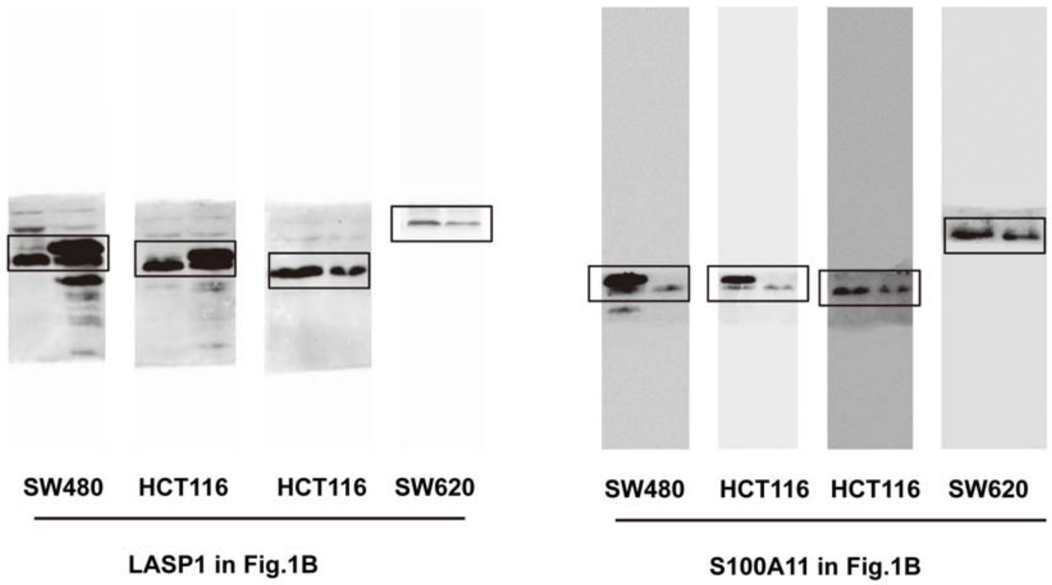

Figure S4, related to Figure 1. The full-length blots/gels including the key data presented in Fig. 1.

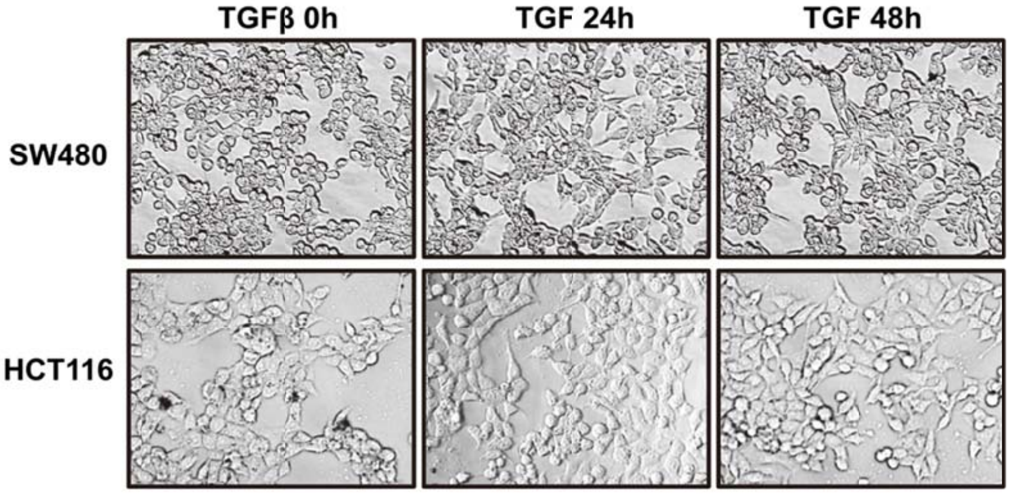

Figure S5, related to Figure 2. The morphology of indicated cells in response to the treatment with TGFβ or control for 24 or 48 hours was observed under an inverted microscope.

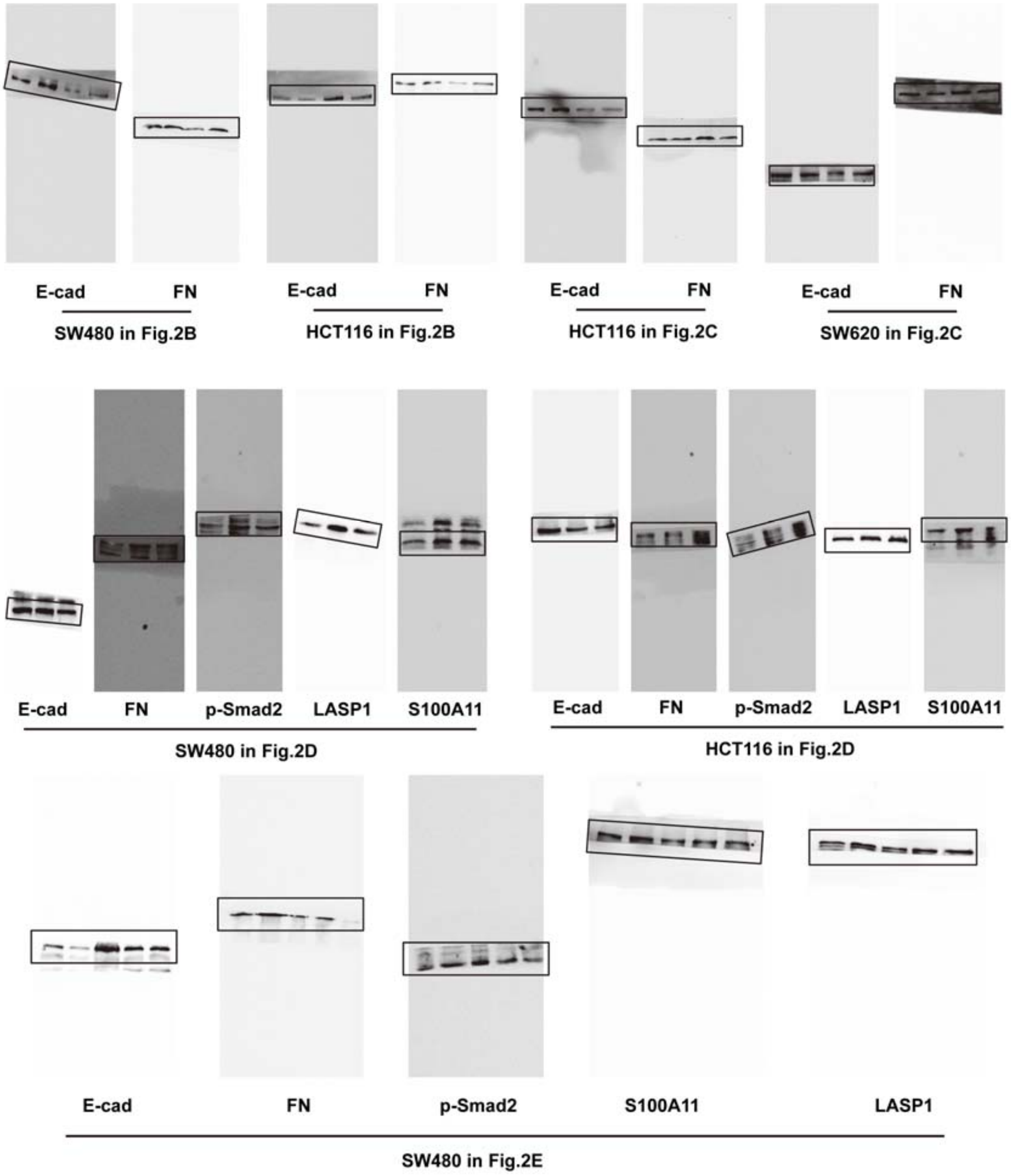

Figure S6, related to Figure 2. The full-length blots/gels including the key data presented in Fig. 2.

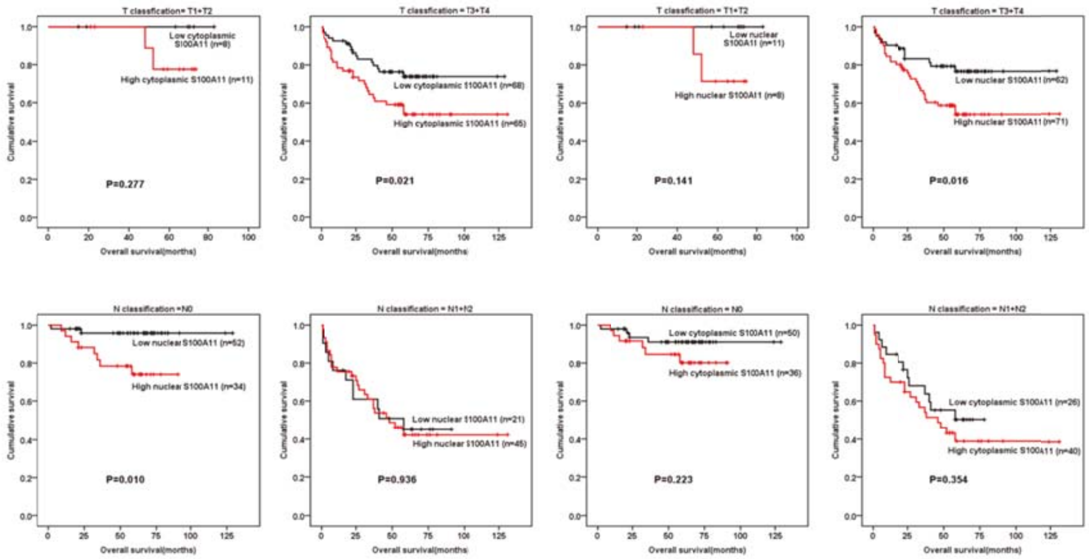

Figure S7, related to Figure 3. Overall survival curves for CRC patients stratified by the S100A11 expression level, according to T classification and N classification. In the T3 and T4 classification tumor subgroups, patients with low expression of cytoplasmic or nuclear S100A11 had significantly better overall survival than patients with high S100A11 expression. In the N0 classification tumor subgroups, patients with low S100A11 nuclear expression had significantly better overall survival than patients with high low S100A11 nuclear expression.

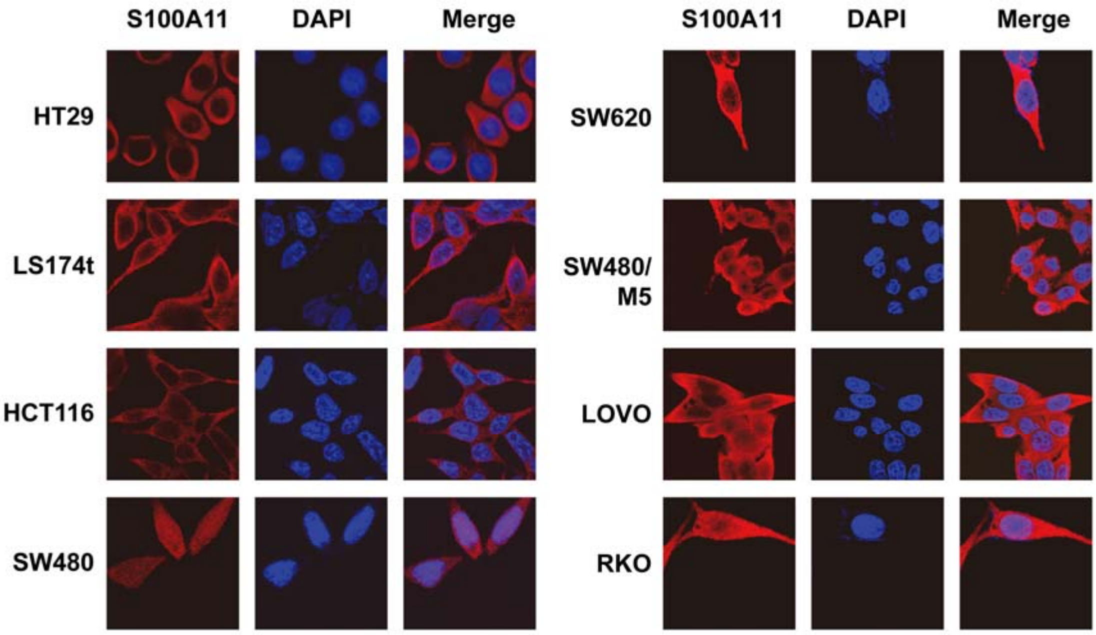

Figure S8, related to Figure 3. The subcellular localization of S100A11 in indicated cells was assessed by immunofluorescence staining.

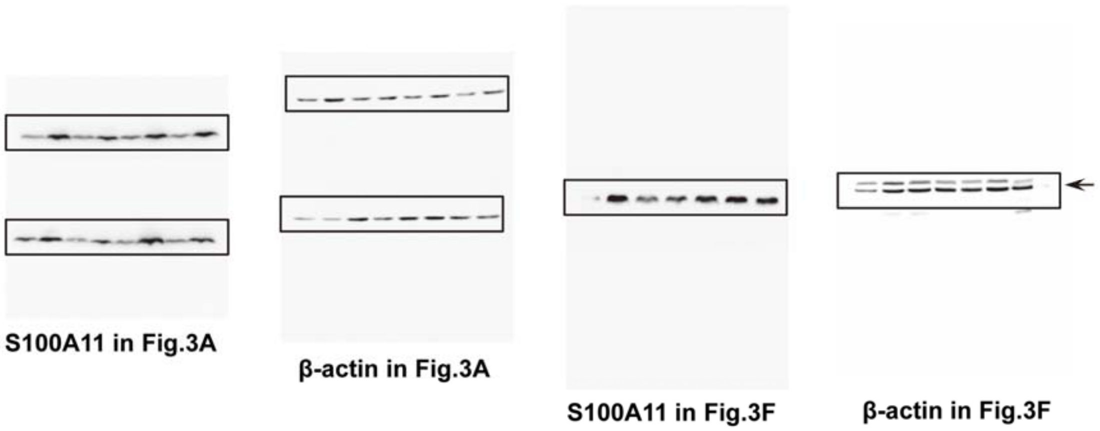

Figure S9, related to Figure 3. The full-length blots/gels including the key data presented in Fig. 3.

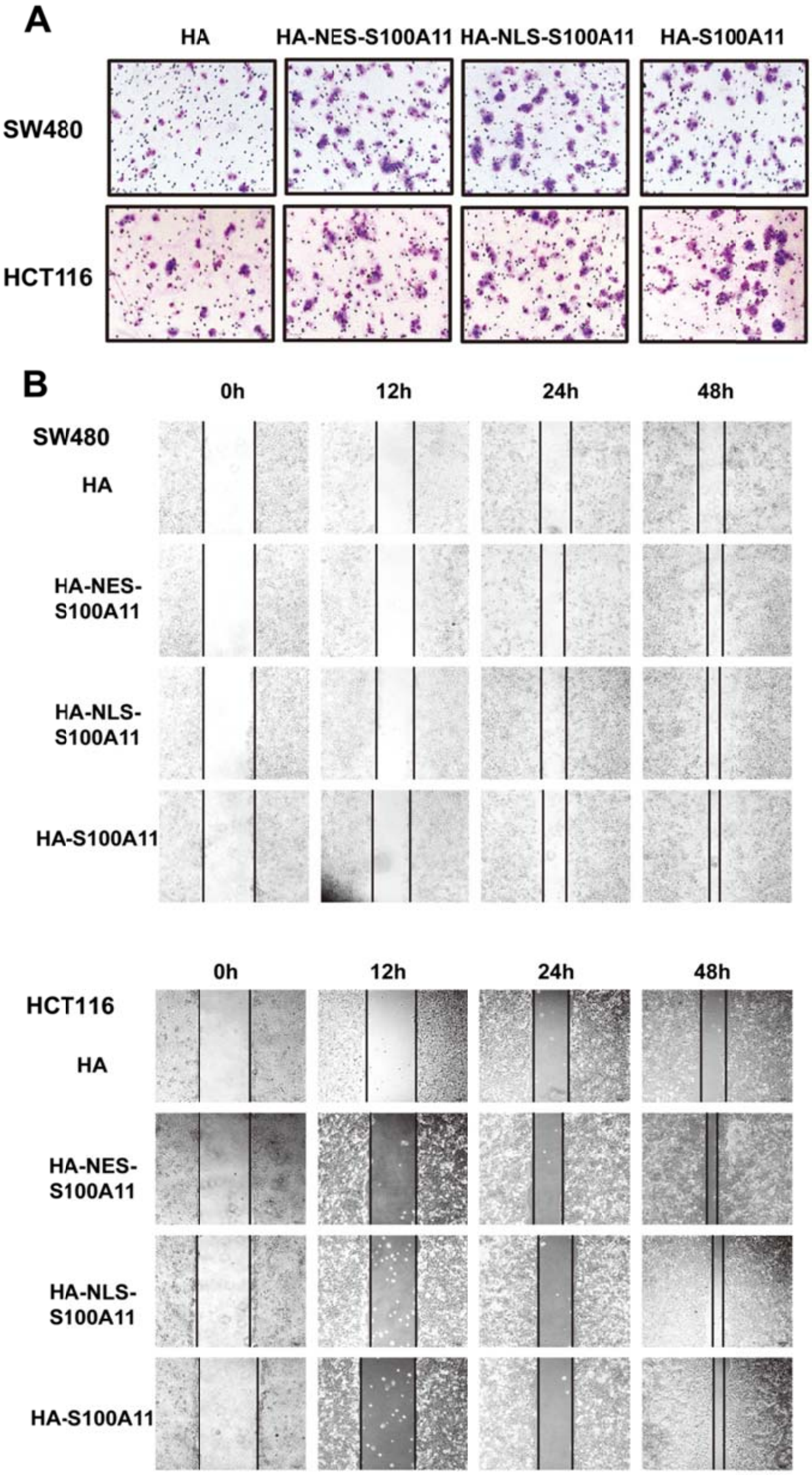

Figure S10, related to Figure 4. The representative figures of transwell (A) and wound healing assay (B) for SW480 and HCT116 cells that were infected with HA, NES-HA-S100A11, NLS-HA-S100A11 and HA-S100A11 vector, respectively.

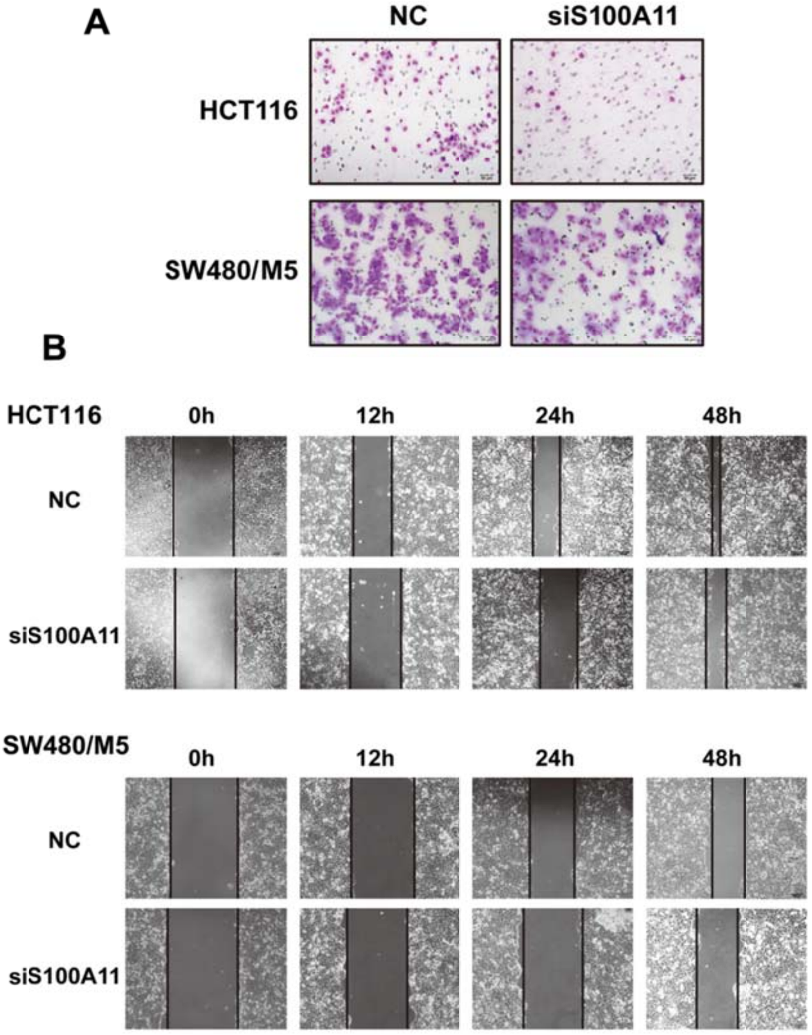

Figure S11, related to Figure 4. The representative figures of transwell assay(A) and wound healing assay (B) for HCT116 and SW480/M5cells that were infected with S100A11 siRNA.

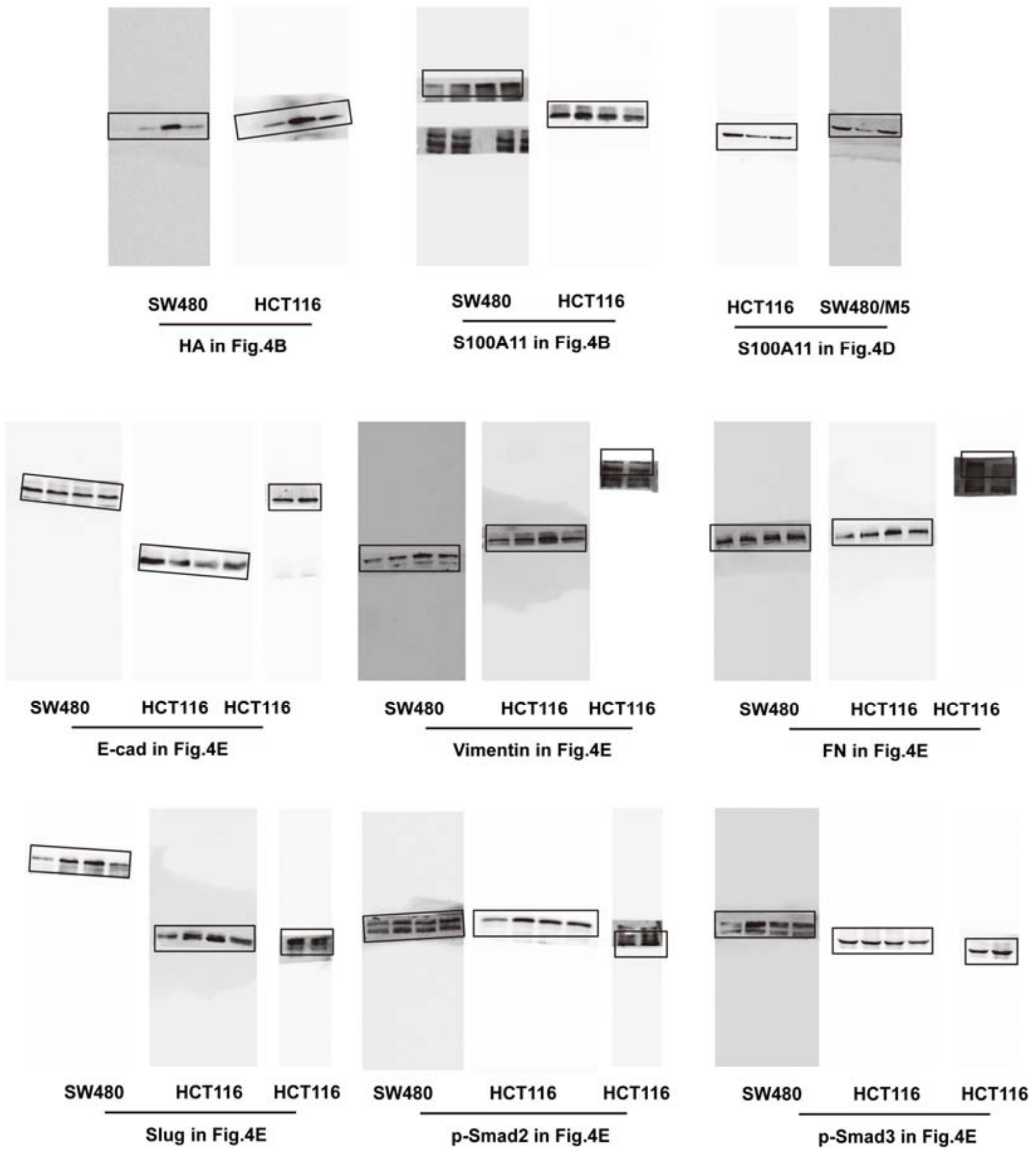

Figure S12, related to Figure 4. The full-length blots/gels including the key data presented in Fig. 4.

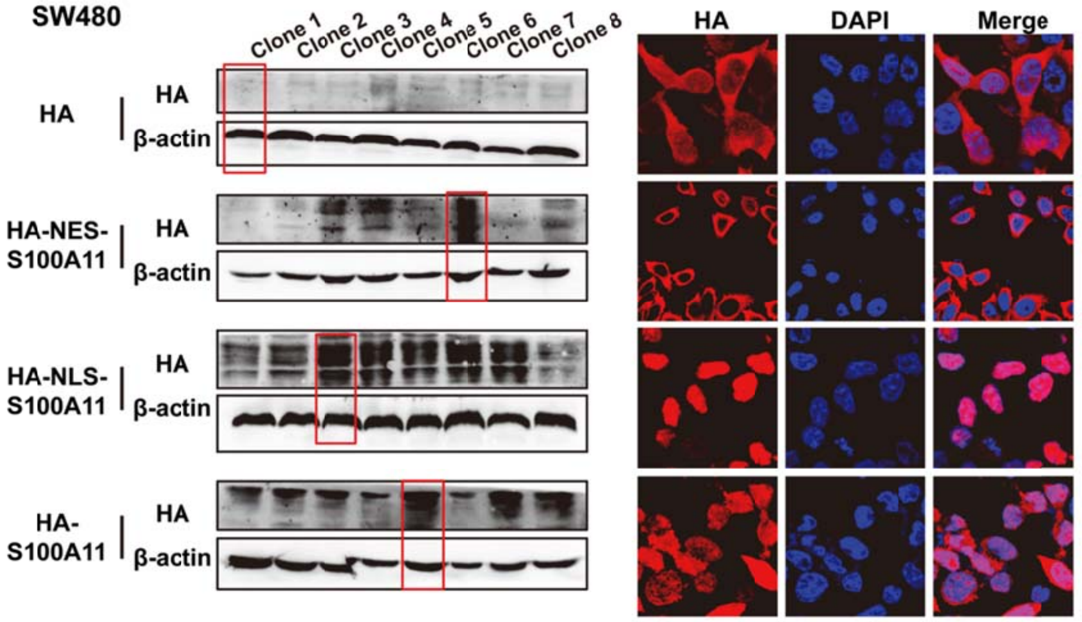

Figure S13, related to Figure 5. SW480 CRC cell lines with stable S100A11 overexpression targeting to cytoplasm and nucleus were established and successfully validated by western blot and immunofluorescence assay. Cell clones with relatively highest transfection efficiency (red box) were selected for *in vivo* animal experiments.

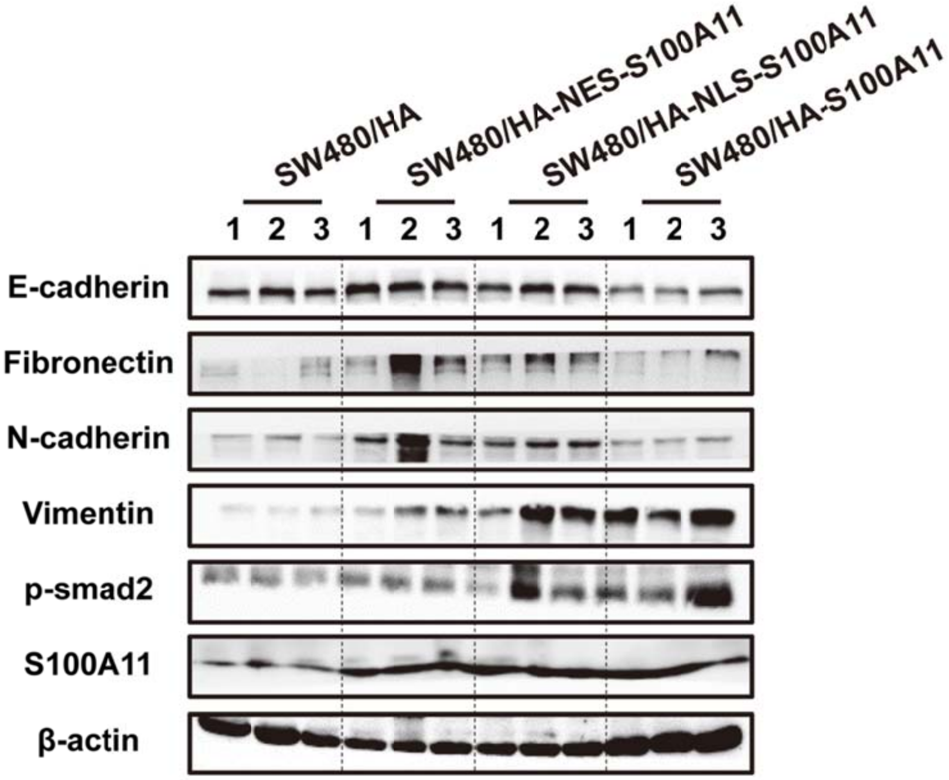

Figure S14, related to Figure 5. Western blot analysis of E-cadherin, fibronectin, N-cadherin, vimentin, p-smad2 and S100A11 expression in subcutaneous tumors.

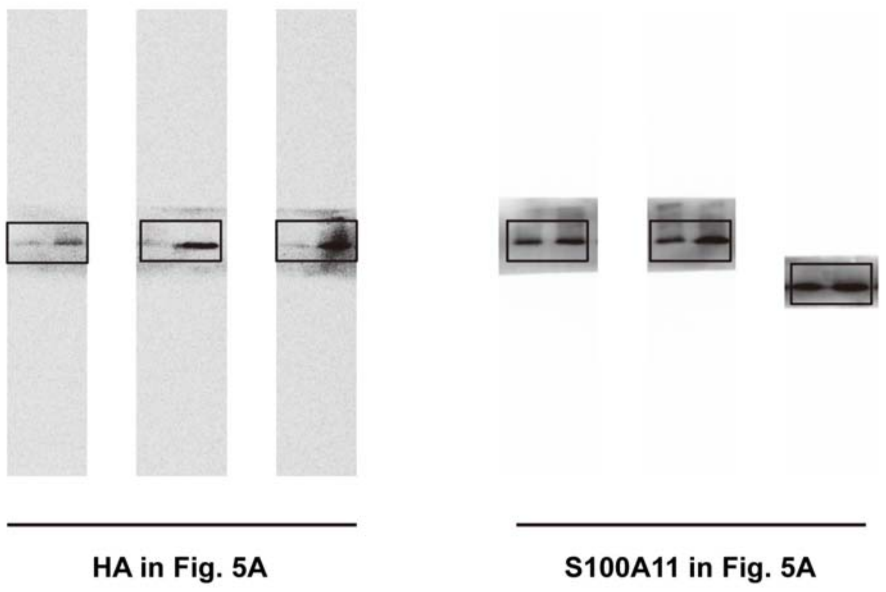

Figure S15, related to Figure 5. The full-length blots/gels including the key data presented in Fig. 5.

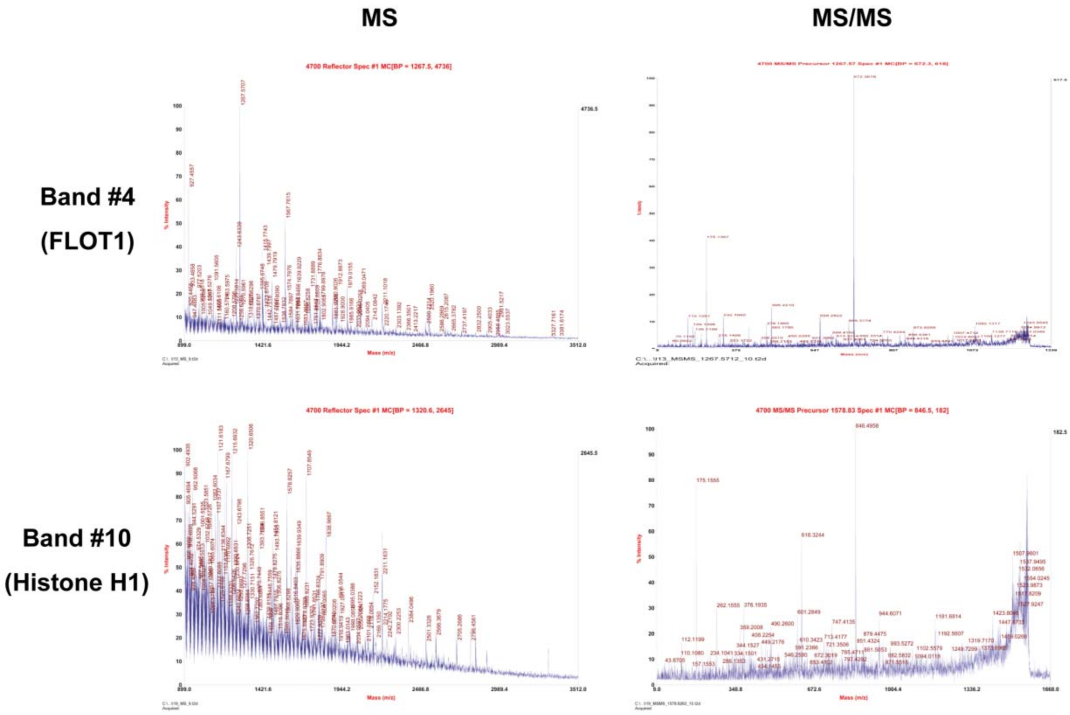

Figure S16, related to Figure 6. MS spectrum obtained from band 4 and 10 after trysin digestion and MS/MS spectrum of the doubly charged precursor ion.

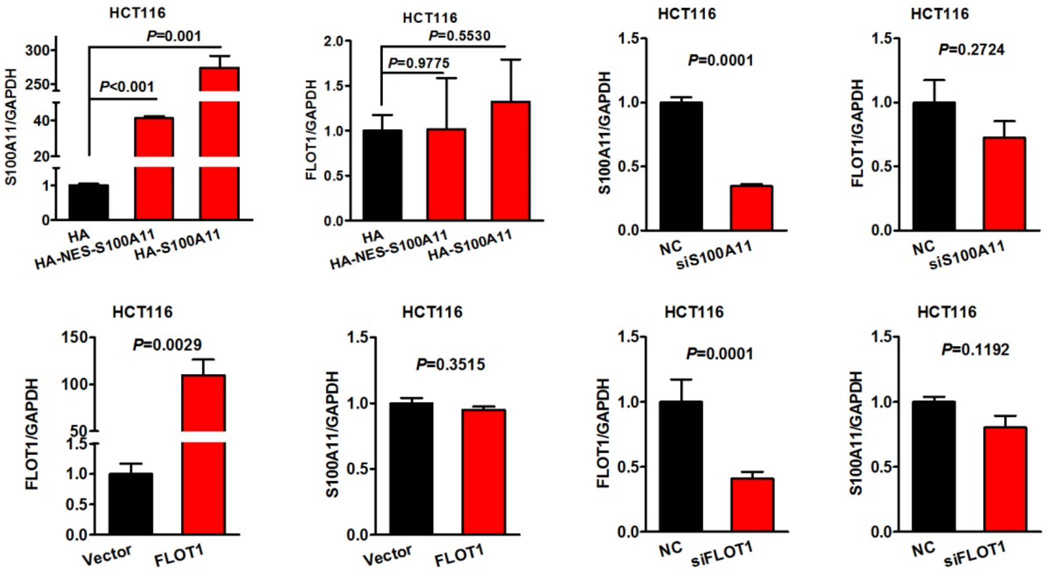

Figure S17, related to Figure 6. The effects between FLOT1 and S100A11 gene at mRNA expression level. RT-PCR assay was performed to detect the expression of FLOT1 and S100A11 mRNA in indicated cells.

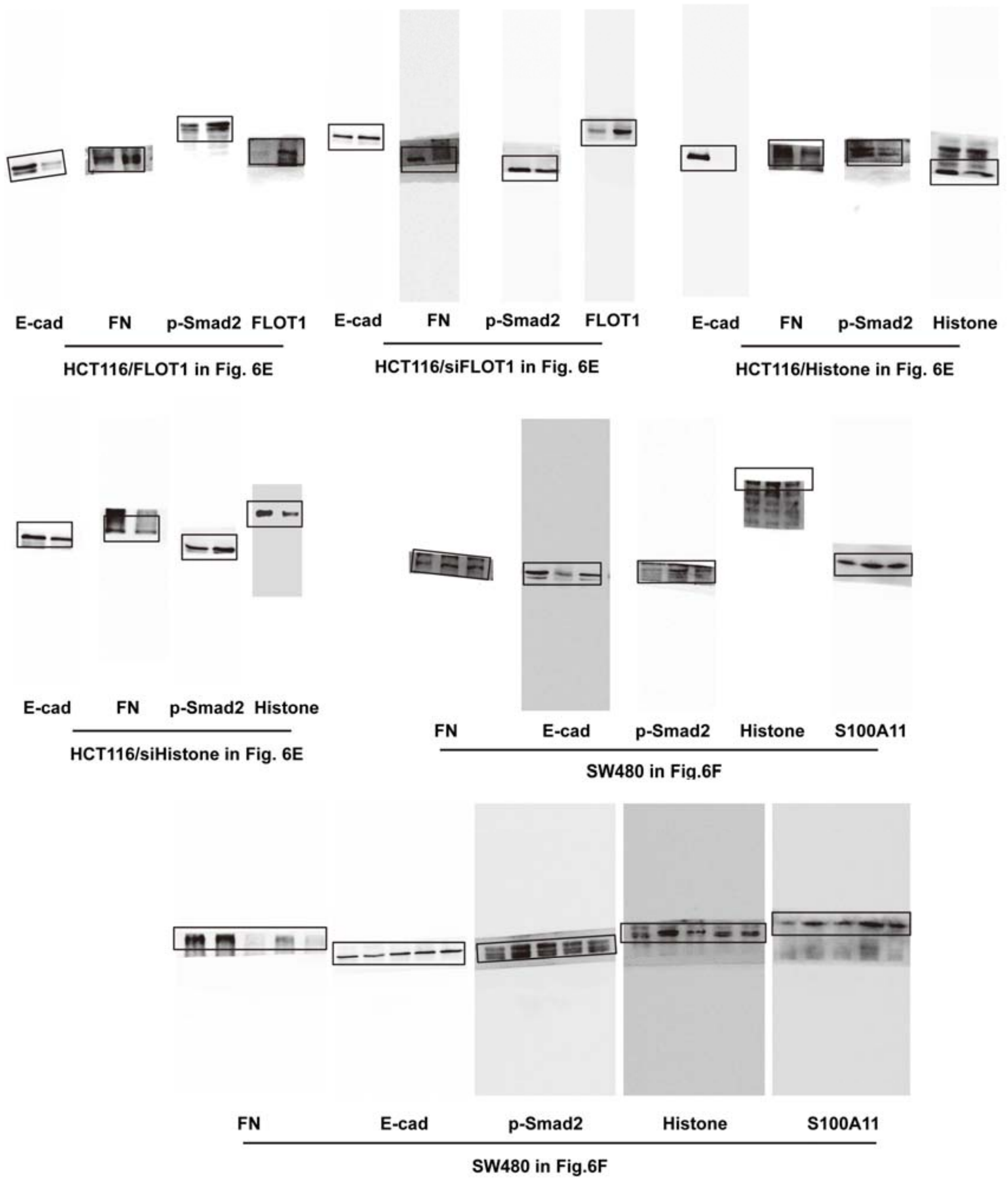

Figure S18, related to Figure 6. The full-length blots/gels including the key data presented in Fig. 6.

Supplementary Table S1. RT-PCR primer sequences for human genes

Gene	Forward primer	Reverse primer	Product length
**S100A11**	GCATCGAGTCCCTGATTGCT	GGGTCCTTCTGGTTCTTTGTGA	133bp
**FLOT1**	CCAGCCTGAACCATGTTTTT	CCATGGCGAGTGTAAACCTT	191bp
**LASP1**	GCAACAGAGTGAGCTCCAGAG	TGAAACCTTTGCCCTTGTTC	70bp
**GAPDH**	GGAGCGAGATCCCTCCAAAAT	GGCTGTTGTCATACTTCTCATGG	197bp

Supplementary Table S2. siRNA sequences used for transfection

Gene name	Sense 5' to 3'	Antisense 5' to 3'
**LASP1**	UUCUCCGAACGUGUCACGUTT	ACGUGACACGUUCGGAGAATT
**S100A11-1#**	CUGGAAAGGAUGGUUAUAATT	UUAUAACCAUCCUUUCCAGTT
**S100A11-2#**	CUAGCUGCCUUCACAAAGATT	UCUUUGUGAAGGCAGCUAGTT
**FLOT1**	CUCAAUGUCAAGAGUGAAATT	UUUCACUCUUGACAUUGAGGG
**Histone H1(0)**	CCCAAGUAUUCAGACAUGATT	UCAUGUCUGAAUACUUGGGTT
**Negative control (NC)**	UUCUCCGAACGUGUCACGUTT	ACGUGACACGUUCGGAGAATT

Supplementary Table S3. Correlation between the clinicopathological features and S100A11 expression

Characteristics	Cytoplasmic S100A11 expression						Nuclear S100A11 expression					
	Negative	Positive	*P* value	Low	High	*P* value	Negative	Positive	*P* value	Low	High	*P* value
Normal	36	33	0.000*	46	23	0.021*	33	36	0.033*	43	26	0.049*
Cancer	27	125		76	76		52	100		73	79	
**Gender**												
Male	13	81	0.106	44	50	0.316	30	64	0.448	42	52	0.293
Female	14	44		32	26		22	36		31	27	
**Age(years)**												
<50	4	18	0.579	11	11	1.000	6	16	0.458	9	13	0.470
≥50	23	107		65	65		46	84		64	66	
**Tumor site**												
Colon	11	66	0.256	39	38	0.871	25	52	0.646	35	42	0.520
Rectum	16	59		37	38		27	48		38	37	
**Tumor size (cm in diameter)**												
<5	14	71	0.639	39	46	0.253	25	60	0.160	38	47	0.356

≥5	13	54		37	30		27	40		35	32	
**Differentiation**												
Good/Moderate	24	112	1.000	69	67	0.597	49	87	0.265	68	68	0.191
Poor	3	13		7	9		3	13		5	11	
**T classification**												
T1+T2	3	16	1.000	8	11	0.462	6	13	0.796	11	8	0.357
T3+T4	24	109		68	65		46	87		62	71	
**N classification**												
N0	18	68	0.244	50	36	0.022*	36	50	0.023*	52	34	0.000*
N1+N2	9	57		26	40		16	50		21	45	
**M classification**												
M0	23	113	0.488	70	66	0.290	48	88	0.412	67	69	0.373
M1	4	12		6	10		4	12		6	10	
**Recurrence**												
Negative	18	76	0.696	55	39	0.033*	37	57	0.303	54	40	0.043*
Positive	6	31		14	23		11	26		14	23	

* Statistically significant ($P < 0.05$).

Supplementary Table S4. Univariate and multivariate analyses of individual parameters for correlations with overall survival rate: Cox proportional hazards model

**Variables**	**Univariate**			**Multivariate**		
	**OR**	**CI(95%)**	***P* value**	**OR**	**CI(95%)**	***P* value**
**Gender**	2.545	1.259-5.144	0.009*	2.422	1.187-4.943	0.015*
**Age**	0.513	0.184-1.433	0.203			
**Tumor site**	0.691	0.382-1.249	0.221			
**Tumor size**	0.559	0.310-1.006	0.052			
**Differentiation**	0.251	0.127-0.496	0.000*	0.309	0.150-0.636	0.001*
**T classification**	0.288	0.070-1.190	0.086			
**N classification**	0.176	0.087-0.355	0.000*	0.304	0.140-0.663	0.003*
**M classification**	0.157	0.082-0.301	0.000*	0.314	0.155-0.633	0.001*
**Cytoplasmic S100A11 overexpression**	0.498	0.271-0.918	0.025*	0.936	0.401-2.182	0.878
**Nuclear S100A11 overexpression**	0.416	0.218-0.794	0.008*	0.657	0.302-1.429	0.289

Abbreviations: OR, Odds ratio; CI, Confidence interval.

* Statistically significant ($P < 0.05$).

Supplementary Table S5. The differential protein bands identified by MS.

Band Number	Protein Name	Protein Accession Number	Protein MW	Protein PI	Protein Score	Protein Score C.I.%
**1**	Inositol polyphosphate 5-phosphatase OCRL-1	sp|Q01968|OCRL_HUMAN	104138	6.13	50	79.665
**2**	Septin-9	sp|Q9UHD8|SEPT9_HUMAN	65360.8	9.06	47	59.426
**3**	Probable phospholipid-transporting ATPase IK	sp|O60423|AT8B3_HUMAN	147935.9	7.97	39	0
**4**	Flotillin-1	sp|O75955|FLOT1_HUMAN	47325.6	7.08	56	95.122
**5**	Tetratricopeptide repeat protein 31	sp|Q49AM3|TTC31_HUMAN	57069.2	8.52	33	0
**6**	ATP-dependent DNA helicase 2 subunit 2	sp|P13010|KU86_HUMAN	82652.3	5.55	135	100
**7**	G protein-regulated inducer of neurite outgrowth 2	sp|O60269|GRIN2_HUMAN	47420.4	6.28	39	0
**8**	Keratin, type II cytoskeletal 1	sp|P04264|K2C1_HUMAN	65999	8.15	54	92.785
**9**	Keratin, type II cytoskeletal 8	sp|P05787|K2C8_HUMAN	53671.1	5.52	101	100
**10**	Histone H1t	sp|P22492|H1T_HUMAN	22005.7	11.71	68	99.655
